# Glycomics in Human Diseases and Its Emerging Role in Biomarker Discovery

**DOI:** 10.3390/biomedicines13082034

**Published:** 2025-08-21

**Authors:** Sherifdeen Onigbinde, Moyinoluwa Adeniyi, Oluwatosin Daramola, Favour Chukwubueze, Md Mostofa Al Amin Bhuiyan, Judith Nwaiwu, Tuli Bhattacharjee, Yehia Mechref

**Affiliations:** Department of Chemistry and Biochemistry, Texas Tech University, Lubbock, TX 79409, USA; sonigbin@ttu.edu (S.O.); moadeniy@ttu.edu (M.A.); odaramol@ttu.edu (O.D.); fchukwub@ttu.edu (F.C.); mdmobhui@ttu.edu (M.M.A.A.B.); jnwaiwu@ttu.edu (J.N.); tuli.bhattacharjee@ttu.edu (T.B.)

**Keywords:** glycosylation, glycomics, cancer biomarkers, neurodegenerative disease, immune modulation, glycan biosynthesis, glycoengineering, translational glycobiology

## Abstract

Glycosylation, the enzymatic addition of glycans to proteins and lipids, is a critical post-translational modification that influences protein folding, stability, trafficking, immune modulation, and cell signaling. The vast structural diversity of glycans arising from differences in monosaccharide composition, branching, and terminal modifications such as sialylation, fucosylation, and sulfation underpins their functional specificity and regulatory capacity. This review provides a comprehensive overview of glycan biosynthesis, with a focus on *N*-glycans, *O*-glycans, glycosaminoglycans (GAGs), and glycolipids. It explores their essential roles in maintaining cellular homeostasis, development, and immune surveillance. In health, glycans mediate cell–cell communication, protein interactions, and immune responses. In disease, however, aberrant glycosylation is increasingly recognized as a hallmark of numerous pathological conditions, including cancer, neurodegenerative disorders, autoimmune diseases, and a wide range of infectious diseases. Glycomic alterations contribute to tumor progression, immune evasion, therapy resistance, neuroinflammation, and synaptic dysfunction. Tumor-associated carbohydrate antigens (TACAs) and disease-specific glycoforms present novel opportunities for biomarker discovery and therapeutic targeting. Moreover, glycan-mediated host–pathogen interactions are central to microbial adhesion, immune escape, and virulence. This review highlights current advances in glycomics technologies, including mass spectrometry, lectin microarrays, and glycoengineering, which have enabled the high-resolution profiling of the glycome. It also highlights the emerging potential of single-cell glycomics and multi-omics integration in precision medicine. Understanding glycome and its dynamic regulation is essential for uncovering the molecular mechanisms of disease and translating glycomic insights into innovative diagnostic and therapeutic strategies.

## 1. Introduction

Glycomics is the comprehensive study of glycans (complex carbohydrates) and their biological roles within organisms. Glycans, also known as carbohydrates, are essential biomolecules ubiquitously present on the surfaces of cells and secreted proteins, playing crucial roles in cell–cell communication, immune recognition, and signaling pathways [1,2,3]. The field of glycomics involves analyzing the structure, biosynthesis, interactions, and functions of glycans across various biological systems. It extends beyond merely characterizing the composition of sugars, encompassing the study of glycan–protein, glycan–lipid, and glycan–cell interactions, which collectively define the functional landscape of glycobiology [4,5,6]. The site-specific characterization of glycans on proteins is termed glycoproteomics.

Glycans contribute fundamentally to a diverse range of biological processes, including cellular growth, differentiation, development, and immune system modulation [7,8,9,10,11]. They modulate protein stability and solubility, influence receptor binding, and govern protein localization [12,13,14]. Glycosylation, the process through which glycans attach covalently to proteins and lipids, represents one of the most common and critical post-translational modifications, significantly impacting protein function and cellular signaling [15]. Glycans regulate interactions between pathogens and host cells, mediate inflammatory responses, and facilitate cellular communication, underscoring their broad biological significance [16,17,18].

The importance of glycomics arises from both the structural complexity and functional significance of glycans. Glycans exhibit immense structural diversity resulting from variations in monosaccharide composition, branching patterns, linkages, and modifications such as sialylation, fucosylation, sulfation, and phosphorylation [19,20]. This structural heterogeneity greatly exceeds that of proteins and nucleic acids, presenting unique challenges and opportunities for biological regulation. Such complexity underlies the specificity and selectivity with which glycans mediate their biological roles, influencing cellular recognition processes and ensuring precise biological control in health and disease. Alterations in glycosylation patterns have been increasingly recognized as hallmarks of numerous pathological conditions, including cancer [21,22,23,24,25], infectious diseases [26,27,28,29], autoimmune disorders [30,31,32], neurodegenerative diseases [33,34], and congenital disorders of glycosylation (CDGs) [35]. Aberrant glycosylation affects disease pathogenesis by altering cell signaling, modifying immune recognition, facilitating tumor progression, and influencing inflammation [36]. In cancer, for example, altered glycosylation of cell surface glycoproteins and glycolipids enables tumor cells to escape immune detection, facilitate metastatic potential, and promote drug resistance [37]. Similarly, in neurodegenerative disorders like Alzheimer’s and Parkinson’s, dysregulated glycosylation contributes to protein aggregation, neuronal dysfunction, and chronic inflammation, demonstrating the profound functional impact of glycans in disease pathology [33,34].

Key mechanisms linking glycosylation to disease include changes in glycan biosynthetic enzyme expression, dysfunction of glycan-processing pathways, and alterations in glycan recognition by lectins and antibodies. Glycan modifications like sialylation and fucosylation significantly impact immune cell function, pathogen recognition, and inflammatory responses [38,39]. For instance, increased sialylation on tumor cell surfaces inhibits immune activation by interacting with immune checkpoints, whereas aberrant fucosylation patterns in autoimmune conditions affect immune tolerance mechanisms [40]. These mechanisms illustrate the multifaceted role of glycosylation in modulating disease onset and progression.

The primary objective of this review is to comprehensively examine glycomics within physiological and pathological contexts, highlighting essential roles of glycans in health, disease development, and progression. This review aims to elucidate fundamental concepts in glycobiology, including the biosynthesis, structural diversity, and key biological functions of glycans, as well as their critical involvement in maintaining homeostasis and regulating developmental processes. Additionally, we will explore glycomic alterations across multiple disease states, including cancer, neurodegenerative diseases, cardiovascular diseases, autoimmune and inflammatory disorders, and infectious diseases, providing insights into the underlying molecular mechanisms linking glycosylation abnormalities to disease pathogenesis. Furthermore, the review will critically analyze current translational applications of glycomics, focusing on the potential use of glycans as diagnostic biomarkers and therapeutic targets, and the emerging field of glycoengineering for precision medicine. Finally, we will address existing analytical and methodological challenges, discuss emerging technological advances such as single-cell glycomics and integrative multi-omics approaches, and propose future research directions intended to enhance clinical translation and facilitate the integration of glycomics into personalized medicine strategies.

## 2. Fundamentals of Glycobiology and Glycan Function in Health

### 2.1. Biosynthesis and Structural Diversity of Glycans

**Figure 1:**
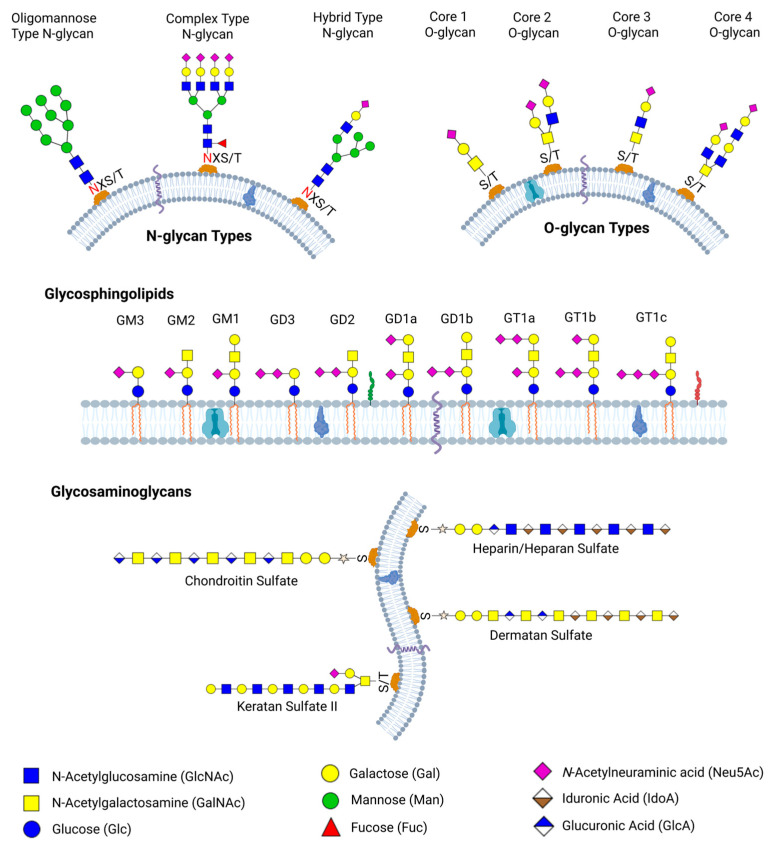
Schematic representation of major classes of glycans found on mammalian cell surfaces. *N*-glycans, including oligomannose, complex, and hybrid types, are attached to asparagine residues within consensus sequence N-X-S/T, while *O*-glycans are linked to serine or threonine residues. Glycosphingolipids are glycolipids with diverse glycan headgroups, including gangliosides such as GM1, GD1, and GT1 variants, which are anchored to membrane via ceramide tails. Glycosaminoglycans such as chondroitin sulfate, dermatan sulfate, heparan sulfate, keratan sulfate II, and heparin are linear polysaccharides attached to proteoglycans and play critical roles in cellular signaling and matrix organization. Legend denotes common monosaccharide residues found in each glycan type.

Glycan synthesis involves a series of enzymatic activities at different locations within the cells, with each enzyme contributing step-by-step to the formation of the final glycan structure [41]. This gives rise to various glycoforms, including glycolipids, glycosaminoglycans, proteoglycans, and glycoproteins, which contain both *N*-linked and *O*-linked glycans. Schematic representations of some examples of *N*-linked glycans, *O*-linked glycans, glycosphingolipids, and glycosaminoglycans are shown in Figure 1. The specific combination of enzymes present in a cell type, along with their regulated activities in space and time, contributes to the vast structural and functional diversity of glycans observed across tissues and physiological conditions.

#### 2.1.1. *N*-Glycan Biosynthesis and Structural Diversity

*N*-glycans are complex carbohydrates that are covalently linked to proteins at the nitrogen atom of an asparagine residue via an *N*-glycosidic bond. Protein *N*-glycosylation can be found in both eukaryotes and prokaryotes [42]. In eukaryotes, glycosylation begins at the membrane of the endoplasmic reticulum (ER) and is further processed in the Golgi apparatus [20]. The synthesis starts with the assembly of glucose and mannose sugar on a lipid-like polyisoprenoid molecule known as dolichol-phosphate (Dol-P). This forms a branched structure of Glc3Man9GlcNAc2 which is transferred to an asparagine residue within a protein sequence motif Asn-X-Ser/Thr where X represents any amino acid except proline through the action of oligosaccharyltransferase (OST). The transferred *N*-glycan undergoes trimming by the glucosidase and mannosidase enzymes, resulting in a more processed *N*-glycan structure, Man7GlcNAc2. The glycosylated protein undergoes chaperone-guided protein folding before being transported to the Golgi apparatus for additional *N*-glycan modification. In the Golgi apparatus, different glycosyltransferases add specific sugar molecules such as galactose (Gal), *N*-acetylglucosamine (GlcNAc), and sialic acid to the *N*-glycan structure that is being formed [42,43,44]. The final composition or microheterogeneity of the *N*-glycan is influenced by the availability of glycoprotein substrates, the expression levels and cellular location of the essential glycosyltransferases, and the supply of the activated sugar donors [45]. In prokaryotes, *N*-glycosylation is less prevalent than in eukaryotes and occurs at the plasma membrane within the periplasmic space. *N*-glycan synthesis in bacteria can occur in two ways. The first involves attaching individual monosaccharide units to certain amino acid residues by soluble proteins within the cytoplasm. The second is similar to eukaryote’s *N*-glycosylation process. Monosaccharide units are first assembled on Dol-P. However, the Dol-p are more saturated than those of eukaryotes. The *N*-glycans are assembled on the cytoplasmic side of the membrane, then flipped to the outer membrane surface before being transferred to the proteins [46,47].

Structural diversity in *N*-glycan arises from the different enzymatic activities involved in the *N*-glycan biosynthesis in the ER and Golgi apparatus. Despite the few known monosaccharides that make up *N*-glycans, their order, conformational variation, as well as branching, give rise to enormous isomers that impact variation in protein structure and functions [43]. *N*-glycans are categorized into three main types based on their structural modifications. The high mannose (or oligomannose) *N*-glycans, which have several mannose units linked to the core structure, the complex *N*-glycans, which include a variety of sugars such as *N*-acetylglucosamine, mannose, galactose, fucose, sialic acid, and the hybrid *N*-glycans, which combine elements of both high-mannose and complex features. All three *N*-glycan types have the core structure GlcNAc2Man3. The differences in the monosaccharide composition on each *N*-glycosylation site give rise to microheterogeneity [48]. Branching in *N*-glycan, such as tri-antennary, tetra-antennary, and bisecting *N*-glycan, occurs by enzymatic addition of GlcNAc to the α1-3 or α1-6 or core β-mannose of the *N*-glycan core by different glycosyltransferases (GlcNAcT-III, GlcNAcT-IV, GlcNAcT-V) in the Golgi apparatus [49]. This adds to the diversity observed in *N*-glycan structures. Other modifications such as fucosylation are also viable and diverse with respect to the number of fucose attached to the *N*-glycan structure. Diversity in *N*-glycan can be further driven by the presence of certain pathogens or variation in the amount of enzymes present during biosynthesis. This is implicated in various diseases and cancers [50].

#### 2.1.2. *O*-Glycan Biosynthesis and Structural Diversity

*O*-glycans, also referred to as mucin-type glycans, are attached to proteins through the hydroxyl group of serine (Ser) or threonine (Thr) residues. Unlike *N*-glycans, their synthesis occurs in the Golgi apparatus through the stepwise addition of sugars directly on the protein. The process is initiated by polypeptide GalNAc-transferase, which facilitates the transfer of *N*-acetylgalactosamine (GalNAc) onto the hydroxyl side chain of either a serine or threonine amino acid within the protein backbone [51]. This forms the fundamental building block of *O*-glycan. Additional sugars, including galactose (Gal), *N*-acetylglucosamine (GlcNAc), fucose (Fuc), and sialic acid (Sia), are subsequently incorporated. *O*-glycosylation is directed by the distinct substrate preferences of glycosyltransferases and sulfotransferases. These enzymes are arranged in a specific order within the Golgi apparatus: early-acting glycosyltransferases are located in the cis-Golgi, intermediate ones in the medial-Golgi, and enzymes that add terminal sugars such as sialic acid are found in the trans-Golgi. Disruption in the spatial arrangement of these enzymes can prevent the synthesis of essential intermediates or hinder further modifications by prematurely forming terminal structures. Such alteration may contribute to the abnormal glycosylation observed in cancer [52,53].

In terms of structural diversity, variation in *O*-glycan structures arises from differences in their core structures and subsequent modification. The length of *O*-glycan may vary from a single GalNAc unit to chains containing over 20 monosaccharide units. There are about four different *O*-glycan core structures, such as GalNAc-Gal (core 1), GlcNAc-GalNAc-Gal (core 2), GalNAc-GlcNAc (core 3), and GalNAc-GlcNAc-GlcNAc (core 4). Core 1 is a Gal residue covalently attached to a GalNAc through a β 1-3 glycosidic linkage. This is formed by the actions of a T-synthase enzyme and can be further modified by sialylation or fucosylation. The Core 2 involves the addition of a GlcNAc to the GalNAc residue of Core 1 glycan through a β1-6 linkage. This allows for branching and more complex *O*-glycan structures. Core 3 structures are less common than Core 1 and Core 2 structures. It is formed by adding GlcNAc to a GalNAc residue through a β 1-3 glycosidic linkage. Addition of another GlcNAc to the GlcNAc residue of Core 3 through a β 1-6 linkage, forms a Core 4 structure. *O*-glycan structures are linear or bi-antennary and exhibit less branching compared to *N*-glycans [54]. In tumor cells, a single GalNAc linked to serine or threonine is found at increased levels, which hints that the addition of more monosaccharides is blocked by cancer cells [53,55]. Most *O*-glycans are negatively charged because of the presence of sialic acid and sulfated monosaccharides at the terminal. These terminal monosaccharides play important roles in cell adhesion and protein stability [56].

#### 2.1.3. Glycosaminoglycans Biosynthesis and Structural Diversity

Glycosaminoglycans (GAGs) are essential heteropolysaccharides, such as hyaluronan, heparin, and chondroitin sulfate, that are involved in various biological processes within multicellular organisms. They are known to be present in the extracellular matrix, in cells, and on cell surfaces, binding to various proteins and regulating cell-pathogen interactions, cell adhesion, cell proliferation, cell differentiation, and blood coagulation [45,57]. GAGs are long anionic, and hydrophilic sugars composed of disaccharide units of *N*-acetylated or *N*-sulfated glucosamine, uronic acid, or galactose as part of their repeating structure. The polysaccharide chains are larger than other glycan types, with about 80 monosaccharide units in a 20 kDa glycosaminoglycan [58]. GAGs are classified into four types including Hyaluronic acid, Chondroitin sulfate, Heparin/heparan sulfate, and Keratan sulfate [59].

Hyaluronic acid is a non-sulfated linear glycosaminoglycan that is made up of repeating units of *N*-acetylglucosamine (GlcNAc) and glucuronic acid (GlcA), connected by alternating β-1,3 and β-1,4 glycosidic bonds [60]. They are found inside mammalian cells, on the cell surfaces, and throughout extracellular matrix. HA is known to be involved in several functions such as cell proliferation, cell migration, and inflammation [61,62,63]. Unlike other glycosaminoglycans, hyaluronic acid is not heavily modified and can differ based on molecular weight. The higher molecular weight inhibits angiogenesis and exhibits anti-inflammatory properties, while the lower molecular weight triggers angiogenesis and inflammation [64]. The biosynthesis of hyaluronic acid is mediated by three different isoenzymes: Hyaluronic acid synthases 1,2, and 3 (HAS 1,2, and 3). The hyaluronan synthases mediate the HA chain synthesis by stepwise addition of GlcNAc and GlcA using the cytosolic UDP-GlcNAc and UDP-GlcA as precursors. The high molecular weight HA is synthesized by HAS 1 and HAS 2, while HAS 3 forms the low molecular weight HA [65,66]. Hyaluronic acids are not attached to proteins or lipids; they are made as free glycans [67].

Chondroitin sulfate is a sulfated glycosaminoglycan found on cell surfaces and within the extracellular matrices [68]. Chondroitin sulfate and its isomer dermatan sulfate consist of repeating disaccharide units, where glucuronic acid (GlcA) or iduronic acid (IdoA) are connected to GalNAc through β-1,3 and β-1,4 glycosidic bonds. They are modified by sulfonic acid at the hydroxyl group of GlcA C-2 and the hydroxyl group of GalNAc C-4/C-6 [69]. The biosynthesis of CS takes place in the endoplasmic reticulum and Golgi compartment. The process starts with the formation of GlcAβ1–3Galβ1–3Galβ1–4Xylβ1–O-Ser (a tetrasaccharide linked to protein), which is the GAG-protein linkage region. This is attached to specific serine residues in various core proteins [70]. The tetrasaccharide is assembled step by step through the addition of specific monosaccharides, including one xylose, two galactose molecules, and one glucuronic acid, catalyzed by xylosyltransferase, β1,4-galactosyltransferase I, β1,3-galactosyltransferase II, and β1,3-glucuronyltransferase I, respectively [71,72,73,74]. The initiation of chondroitin backbone synthesis begins with the transfer of GalNAc to the nonreducing end of the GlcA in the GAG-protein linkage region, a step carried out by GalNAc transferase I. This is followed by the alternating addition of GlcA and GalNAc units, catalyzed by GlcA transferase II and GalNAc transferase II, respectively, resulting in the formation of the repeating disaccharide structure characteristic of chondroitin sulfate [70,75]. Chondroitin sulfate is involved in a range of physiological and pathological processes, including cell division, neural plasticity, bone-related diseases, and infections [75,76,77].

Heparin is composed of GlcA or IdoA linked through an α-1,4 glycosidic bond to *N*-acetylglucosamine (GlcNAc). In contrast, Heparan sulfate is made up of GlcA or IdoA linked through a β-1,4 glycosidic bond to *N*-acetylglucosamine. The structures contain sulfonic modification at specific sites [78]. The biosynthesis process of this GAG is similar to that of chondroitin sulfate and dermatan sulfate. It involves first the formation of the GAG-protein linkage region [79]. After the assembly of the tetrasaccharide on the core protein, the heparin/heparan sulfate is subsequently polymerized by the alternative addition of GlcA and GlcNAc. This process is performed by two glycosyltransferase enzymes (EXT1 and EXT2) [80]. Additional modifications occur, including *N*-sulfation, *N*-deacetylation, and the C5 epimerization of GlcA, which involves its conversion to IdoA at specific sites. These modifications are mediated by enzymes such as sulfotransferases (for sulfation), *N*-deacetylase-*N*-sulfotransferases, and uronyl C5 epimerases (for C5 epimerization of GlcA) [80,81]. Heparin and heparan sulfate are present on cell surfaces and within the extracellular matrix, where they are involved in various biological processes, including anticoagulation [82], angiogenesis [83], viral invasion [84], and cell growth/development [85].

Keratan sulfate is a linear structure composed of Galactose (Gal) linked to GlcNAc through β-1,3 and β-1,4 glycosidic bonds. The structures undergo sulfonic modification to the hydroxyl groups at C-6 positions of galactose and *N*-acetyl galactosamine [59,86]. Keratan sulfate chain can be disulfated, monosulfated, and nonsulfated, as well as fucosylated or sialylated. The sulfation of the nonreducing end of GlcNAc allows for the addition of monosaccharide units to the chain, while the sulfation of the nonreducing end of Gal blocks further elongation of the chain. The keratan sulfate chains with up to 50 disaccharides units are known as KS I. They are mostly found in the corneal tissues. The KS II, which occurs frequently in cartilages, is made up of chains with less than 50 disaccharide units. The biosynthesis process of these GAGs is catalyzed by *N*-acetyl glucosaminyl 6-*O*-sulfotransferases and galactosyl 6-*O*-sulfotransferases. The process begins with the synthesis of the poly-*N*-acetyllactosamine backbone, followed by the addition of sulfate groups donated by 3′-phosphoadenyl-5′-phosphosulfate, a high-energy sulfate donor. Sulfation is mediated by specific enzymes, with *N*-acetylglucosaminyl 6-*O*-sulfotransferases adding sulfate groups to *N*-acetylglucosamine units and galactosyl 6-O-sulfotransferases transferring sulfate to galactose residues [87,88,89,90]. Unlike other glycosaminoglycans, keratan sulfate does not have glucuronic acid or iduronic acid in its structure. This gives them a less acidic potential in solution. Keratan Sulfate acts as a signaling and hydrating agent in cartilage tissues and the cornea [88]. They are also involved in inflammation [91], cancer [92], and neural plasticity [93].

#### 2.1.4. Glycolipids Biosynthesis and Structural Diversity

Glycolipids and glycosphingolipids are lipid-linked sugars essential for membrane structure, cell recognition, and signaling. They consist of a hydrophilic sugar head and a hydrophobic lipid tail joined by a β-glycosidic bond. Sugar, such as glucose, galactose, oligosaccharides, or sulfated groups, forms complex structures including gangliosides, glycoglycerolipids, and glycophosphatidylinositols [94,95]. Glycolipid biosynthesis involves sequential enzyme actions across subcellular compartments. In mammalian cells, it begins at the cytosolic side of the endoplasmic reticulum with the formation of dihydroceramide, catalyzed by enzymes from six dihydroceramide synthase genes. The dihydroceramide is subsequently flipped to the luminal side and transported to the Golgi, where sugars like glucose and galactose are added. Complex glycolipids such as gangliosides and globosides are further modified with sialic acids, GalNAc, and other units. The length and saturation of the hydrophobic tail vary between 12 to 26 carbon atoms, depending on the tissue [45,95,96,97]. The variation in glycolipids adds significant diversity to their structures. Their structural composition determines their distinct biophysical properties, their membrane surface localization, and their clustering into glycosynapses. The aberrant expression of glycolipids has been implicated in several lysosomal storage diseases and cancer [98,99]. Glycolipids like GM2, GD2, Globo H, SSEAH, etc. are tumor-associated antigens and are potential targets for cancer treatment [100]. Glycolipid dysregulation is also associated with neuroinflammation, which plays a key role in Parkinson’s disease [101].

### 2.2. Glycosylation and Cellular Function

#### 2.2.1. Protein Folding, Stability, and Trafficking

The occurrence of glycosylation during protein synthesis enables it to play a role in determining the protein folding state, protein folding kinetics, and protein stability [102]. Glycosylation affects not only the protein’s thermodynamic stability but also the structural properties of the folded proteins, influencing their functions and interactions [103]. For *N*-glycosylation, the oligosaccharides are attached to the nitrogen atom of the asparagine residue of the newly synthesized polypeptide chain. The attached *N*-glycan, through the high-mannose segment, which is bulky and hydrophilic in nature, maintains the glycoprotein in solution during the folding process. This prevents the aggregation of polypeptides and promotes proper protein conformation. When glycoproteins are improperly folded, the glucosidase I and II enzymes deglucosylate the originally attached glycan, making the glycoprotein monoglucosylated [104,105]. This modification allows the glycoprotein to interact with calnexin or calreticulin, thereby retaining it within the endoplasmic reticulum. The interaction between the glycoprotein and the lectin (calnexin and calreticulin) allows for deglucosylation and refolding of the protein. Once the proper protein folding is achieved, the proteins are detached from the lectin complex and transported to their cellular destination. This protein folding cycle helps to increase the folding efficiency of glycoproteins and prevent the formation of non-native disulfide bonds that support aggregation [106,107]. The absence of aggregation enables the protein to interact with molecular chaperones that facilitate the folding process [104,107,108]. To better understand the impact of *N*-glycosylation on protein folding, stability, and trafficking, Wujek mutated the asparagine on the *N*-glycosylation sites of the human tripeptidyl-peptidase I enzyme gene (TPP I) with glutamine, preventing glycosylation [109]. The authors found that the mutation at Asn-286 changed the folding of the enzymes, and the mutated enzyme was retained in the endoplasmic reticulum. The mutated enzymes also showed reduced stability and decreased activity upon acidification [109]. Another work by Moharir et al. [110], showed how *N*-glycosylation influences protein folding, trafficking, and function.

#### 2.2.2. Cell–Cell Communication and Immune Response

Mammalian cell surfaces are rich in glycoconjugates like glycolipids and proteoglycans. The extracellular matrix contains glycoproteins like laminin and fibronectin that support cell adhesion and polarity. *N*- and *O*-linked glycans on cell surfaces and secreted proteins are crucial for cell communication, development, and disease [111]. For example, branched *N*-glycans on transmembrane proteins serve as primary ligands for galectins on the surface of mammalian cells. *N*-glycosylation is also essential for cell adhesion. Changes in adhesive molecules’ *N*-glycan structures can impact interactions between cells and the extracellular matrix (ECM), which in turn can impact tumor invasion, migration, and cell adhesion [112]. *N*-glycans featuring β1-6 *N*-acetylglucosamine branching are known to be strongly linked to certain malignant phenotypes, a modification driven by the enzyme *N*-acetylglucosaminyltransferase V (GnT-V). In contrast, *N*-acetylglucosaminyltransferase III (GnT-III), an enzyme that catalyzes *N*-glycan bisection, is considered to inhibit cancer metastasis [49,112,113,114].

Antibody glycosylation affects interactions with Fc receptors, complement activation, and inflammation [31]. A common glycosylation pattern that alters the half-life of antibodies is the *N*-glycosylation of terminal mannose. Therapeutic IgG monoclonal antibodies (mAbs) containing high-mannose glycans in their Fc region are eliminated from the bloodstream more quickly than those with complex glycosylation, which show longer half-lives in vivo [115,116]. Additionally, mAbs with G0 oligosaccharides exhibit slightly faster clearance compared to those with different variable domain structures [117]. However, in contrast to other findings, one study in BALB/c mice reported that G0 IgG has a longer half-life than fully glycosylated forms [31,118]. The researchers found that FUT8-mediated glycosylation stabilizes B7H3 on the tumor cell surface, which in turn contributes significantly to immune evasion by inhibiting T-cell activation and cytotoxic function [21].

*O*-GlcNAcylation is important in promoting inflammatory responses, particularly in macrophages, by modifying critical transcription factors, which are essential for inflammatory gene expression and tissue repair [119,120]. *O*-GlcNAcylation has been shown to alter NF-κB, a crucial regulator of inflammatory signaling in macrophages. A prior work has shown that *O*-GlcNAcylation on S350 altered the NF-κB component c-Rel, which is necessary for its DNA-binding and transactivation function [121]. Additionally, *N*-linked glycosylation negatively regulates CD28 function in T cells, as removing glycosylation enhances its binding to CD80 and strengthens T-cell activation, highlighting glycosylation’s pivotal role in modulating immune signaling [122].

### 2.3. Role of Glycans in Homeostasis and Development

#### Neural Development and Synaptic Plasticity

Although neurons rely on glucose for energy, they are also regulated by other sugars. Over 500 million years of evolution, sugars have gained roles in molecular recognition and signaling, influencing protein folding, trafficking, and stability. Glycans, abundant in the brain, impact processes like learning, memory, development, and response to spinal cord injury [123]. While the precise mechanisms remain unclear, glycoprotein-mediated interactions clearly aid synaptic communication. Advanced 2D MRS has uncovered how fucosylated sugars influence brain function and change with development, aging, and disease [124].

Fucosylated proteins called synapsins regulate neurotransmitter release by anchoring synaptic vesicles to the cytoskeleton, preventing uncoordinated diffusion to the synaptic membrane [123]. PolySia glycans, which are widely expressed during the embryonic and postnatal stages of brain development, are also necessary for synaptic plasticity, cell differentiation, and migration [125]. During development, polySia stimulates cell motility by expanding intercellular space, improves cell migration and axon finding, and encourages repair or regeneration in damaged peripheral and central nervous system tissues because of its hydrophilic nature and strong negative charge [126].

## 3. Glycomic Alterations in Diseases

**Figure 2:**
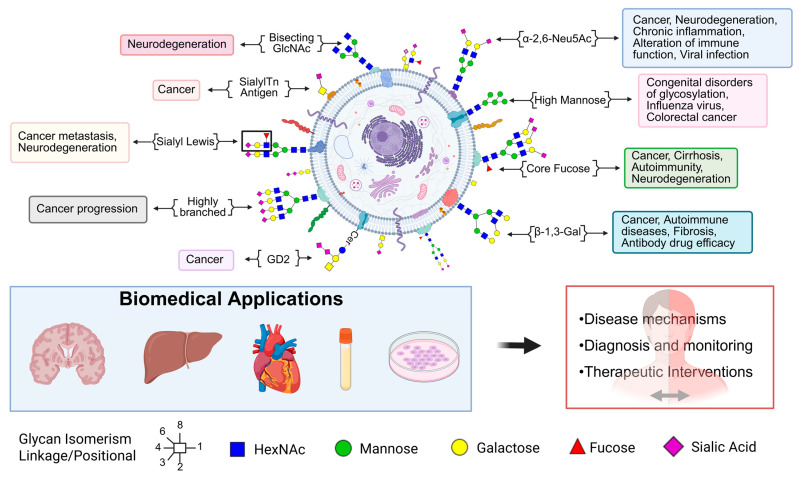
Glycans play critical roles in maintaining health and are involved in the pathogenesis of various diseases. This schematic illustrates the diverse glycan structures present on the cell surface and their associations with specific biological processes and disease states. Structural motifs such as bisecting GlcNAc, sialyl Lewis antigens, high-mannose glycans, core fucose, and highly branched glycans are linked to cancer progression, metastasis, neurodegeneration, autoimmune disorders, and infectious diseases. Altered glycosylation patterns serve as biomarkers and therapeutic targets, influencing immune recognition, cell adhesion, signaling, and drug efficacy. Biomedical applications of glycomics include uncovering disease mechanisms, enhancing diagnosis and monitoring, and guiding therapeutic interventions across tissues and biological systems.

Glycomics plays a critical role in deciphering disease-specific alterations in glycan structures that influence pathogenesis, immune responses, and therapeutic outcomes. Specific glycan motifs such as bisecting GlcNAc and core fucose are implicated in neurodegeneration, cancer, and chronic inflammation [127,128]. Sialyl-Tn antigens and GD2 gangliosides are enriched in tumors, supporting cancer progression and serving as immunotherapy targets [129,130]. Highly branched glycans are linked to cancer invasiveness, while sialyl Lewis antigens are associated with both metastasis and neurodegenerative disorders [131]. High mannose glycans are markers of congenital glycosylation disorders and certain cancers [35]. Additionally, β-1,3-linked galactose structures influence autoimmune activity and drug efficacy. Figure 2 depicts diverse glycan structures and their associations with specific biological processes and disease states. These disease-associated glycan patterns have important biomedical applications, aiding in the mechanistic understanding of diseases, diagnosis and monitoring, and design of targeted therapeutics.

### 3.1. Cancer Glycomics

In cancer cells, changes in glycosylation patterns arise from the disruption of normal regulatory mechanisms governing glycosylation enzymes. This dysregulation alters the structure of *N*-glycans, which can significantly impact various cellular processes [114,132]. Glycosyltransferases, which add sugar moieties to proteins and lipids, and glycosidases, which remove these sugars, often exhibit altered expression and activity in cancer compared to normal tissues [114,133]. For instance, the genes encoding *N*-acetylgalactosaminyltransferases (GALNTs) are frequently overexpressed in cancerous tissues [134,135]. Genetic mutations within genes encoding components of the glycosylation machinery, as well as epigenetic modifications that influence their expression, also contribute significantly to the production of abnormal glycan structures [136]. In addition to altered enzyme levels, the mislocalization of glycosyltransferases within the cellular compartments can also lead to aberrant glycosylation by affecting their access to substrate proteins [137,138]. Moreover, changes in the cellular metabolic environment can impact the availability of activated nucleotide sugars, which serve as the building blocks for glycans, thereby influencing the final glycosylation outcomes.

#### 3.1.1. Aberrant Glycosylation as a Hallmark of Cancer

Cancer cells commonly exhibit a range of specific aberrant glycosylation patterns. One frequent observation is the incomplete synthesis of glycan chains, which leads to the presence of truncated glycans not typically found in healthy cells. A notable example of this is the Tn antigen (GalNAcα1-*O*-Ser/Thr) [22]. Enhanced expression of complex branched *N*-glycans, often regulated by enzymes such as *N*-acetylglucosaminyltransferase V (GnT-V), is another hallmark [23]. The presence of truncated *O*-glycans, including the Tn antigen and its sialylated derivative, Sialyl Tn (STn) antigen (NeuAcα2-6GalNAcα1-*O*-Ser/Thr), is also a common feature in various carcinomas [24,129,139,140]. Furthermore, the overexpression of core fucosylation is frequently observed [21,141]. Altered sialylation, particularly an increase in the overall levels of sialic acid and the upregulation of specific sialyltransferases like ST6GAL1, is also characteristic of cancer and contributes to tumor progression and survival under stress conditions [142,143,144]. Changes in the expression of Lewis antigens (LeX, LeY, LeA, and LeB) and their sialylated forms (sLeX and sLeA), which are essential for cell adhesion and migration, are also prevalent in many cancer types [131,145,146]. Finally, *O*-GlcNAcylation, where a single *N*-acetylglucosamine is added to serine or threonine residues of proteins, is dysregulated in cancer and can affect proteins involved in crucial cellular processes like cell cycle control [147,148].

The unique glycan structures and patterns expressed by cancer cells can enter the bloodstream, presenting opportunities to serve as biomarkers for cancer diagnosis and monitoring. Indeed, a significant number of currently used clinical tumor biomarkers are either glycoproteins themselves or are glycan-related molecules. Examples include alpha-fetoprotein (AFP) for liver cancer [149,150], cancer antigen 125 (CA125) for ovarian cancer [151,152], carcinoembryonic antigen (CEA) for colon cancer [153,154], prostate-specific antigen (PSA) for prostate cancer [155], and carbohydrate antigen 19-9 (CA19-9) for gastrointestinal and pancreatic cancers [156]. Glycomics, the comprehensive analysis of the glycome, holds considerable promise as a research area for identifying novel and more specific cancer biomarkers. Recent studies have successfully identified distinct glycan profiles that can distinguish between cancer patients and healthy individuals for various cancer types, including glioblastoma [157,158,159], meningioma [158], endometrial cancer [160,161,162], breast cancer [163,164], and hepatocellular carcinoma [165,166]. However, the clinical translation of glyco-biomarkers remains challenging because of the inherent abundance, complexity, and dynamic nature of glycan structures, requiring sophisticated analytical methodologies and rigorous validation.

#### 3.1.2. Tumor-Associated Carbohydrate Antigens (TACAs)

Tumor-associated carbohydrate antigens (TACAs) represent a diverse class of carbohydrate structures that are either overexpressed or uniquely found on the surface of cancer cells, distinguishing them from their normal counterparts [167,168]. These antigens can be broadly categorized based on the biomolecules to which they are attached, primarily glycoproteins and glycolipids [169]. Glycoprotein antigens include those found on proteins such as mucins (e.g., MUC1, MUC4, MUC16) and other cell surface and secreted proteins, with examples including the Tn antigen, Thomsen-Friedenreich (T) antigen, and their sialylated forms like sialyl-Tn (STn) [170]. Glycolipid antigens are carbohydrate structures linked to lipid molecules in the cell membrane, encompassing the Globo series antigens (e.g., Globo H), gangliosides (e.g., GD2), and blood group-related antigens such as Lewis antigens and their sialylated derivatives [171].

Several key TACAs have been extensively studied. The Tn antigen (αGalNAc-Ser/Thr) is a simple *O*-linked glycan, often considered a precursor in *O*-glycan biosynthesis. Its expression in cancer typically arises due to a disruption in the normal *O*-glycosylation pathway, frequently linked to the loss of function of the T-synthase enzyme or its molecular chaperone Cosmc [172]. Sialyl Tn antigen (Neu5Acα2-6GalNAcα-O-Ser/Thr) is formed by the addition of sialic acid to the Tn antigen and is synthesized by the sialyltransferase ST6GalNAc I [173]. STn is often associated with poor prognosis in cancer patients [173]. Lewis antigens are fucosylated carbohydrate epitopes built on either type 1 or type 2 lactosamine backbones [174]. Their expression is regulated by various fucosyltransferases and is often elevated in cancer, where they participate in cell adhesion and interactions with selectins [175]. Globo H is a well-characterized hexasaccharide glycolipid initially identified in a breast cancer cell line [176]. It is found in several cancer types and contributes to the tumor microenvironment and disease progression [177]. GD2 ganglioside is a disialoganglioside prevalent on the surface of several neuroectodermal tumors [130]. Mucin 1 (MUC1) is a heavily *O*-glycosylated transmembrane protein found on many epithelial cells. In cancer, MUC1 often exhibits aberrant glycosylation patterns, leading to altered interactions and roles in tumor invasion and metastasis [178].

The prevalence of specific TACAs varies across different cancer types. The Tn and STn antigens are remarkably widespread, found in a majority of human carcinomas, including breast, colon, lung, gastric, and ovarian cancers, and show high expression in esophageal adenocarcinoma [179]. Lewis antigens exhibit cancer-type specific prevalence; for instance, LeA and LeB are commonly found in pancreatic cancer [180], while LeY and its extended forms are highly specific markers for colon cancer malignancy [181]. Globo H is abundant in a broad range of cancers, including breast, ovarian, lung, gastric, prostate, pancreatic, endometrial, and liver cancers [182,183]. GD2 is particularly prevalent in neuroblastoma, melanoma, small-cell lung cancer, glioma, and various sarcomas [130]. CA19-9 (sialyl-Lewis A) is a well-established serum biomarker for digestive system carcinomas, especially pancreatic cancer [184]. Furthermore, specific *O*-glycans with terminal sialyl-LewisX/A or α2-3-linked sialylation have been identified with high specificity in colorectal cancer [185]. Some TACAs are widely expressed across cancers, reflecting common glycosylation changes and offering broad therapeutic potential. Others show cancer-specific patterns, making them useful for targeted diagnosis and treatment. TACAs like Globo H also actively shape the tumor microenvironment, contributing to cancer progression.

#### 3.1.3. Aberrant Glycosylation in Cancer Metastasis, Immune Evasion, and Therapy Resistance

##### Role of Aberrant Glycosylation in Cancer Metastasis

Aberrant glycosylation plays a pivotal role in the complex process of cancer metastasis by influencing crucial cellular events such as cell adhesion, migration, and invasion [132]. Altered glycosylation from dysregulated glycosyltransferases results in abnormal membrane glycans that promote cancer progression. TACAs, arising from these changes, play key roles in metastasis. The ability of cancer cells to attach to one another and to the surrounding extracellular matrix is fundamental to the development of primary tumors and the dissemination of metastatic cells. Aberrant glycosylation profoundly affects cell–cell interactions. For instance, increased branching of *N*-glycans, a process regulated by GnT-V, can alter the function of *N*-cadherin, resulting in a loss of cellular adherence and promoting tumor invasion [186]. Conversely, the removal of certain *N*-glycans from E-cadherin has been shown to enhance the interaction within the cadherin-catenin complex, leading to a stabilization of cell–cell adhesion. Elevated *O*-GlcNAcylation of β-catenin has been proven to enhance the expression of E-cadherin and its subsequent movement into the nucleus, ultimately triggering invasion and metastasis in colorectal cancer [187]. In ovarian cancer, the silencing of the Golgi mannosidase MAN1A1 impairs the *N*-glycosylation of ALCAM, which in turn reduces tumor cell aggregation, adhesion, and movement [188].

Altered glycosylation also significantly promotes the migration and invasion of cancer cells, key steps in the metastatic cascade. The modification of the *N*-glycan structure β1,6-GlcNAc at the Asn-554 site of E-cadherin has been shown to inhibit its biological functions in cell–cell adhesion [189]. Conversely, the inhibition of α1,6 fucosyltransferase improves E-cadherin’s function in cell–cell adhesion, leading to a reduction in tumor invasion in lung cancer [190]. Interestingly, breast cancer cells deficient in FUT8 exhibited reduced cell migration and invasion due to diminished fucosylation of E-cadherin and inhibition of integrin-mediated FAK signaling [191]. Aberrant expression of the glycosyltransferase GALNT3 leads to elevated *O*-glycosylation of the mucin MUC1, which enhances the stability of the E-cadherin and the β-catenin complex, which support ovarian cancer cell growth and movement.

TACAs, often arising from aberrant glycosylation, are key players in metastasis by enabling interactions with elements of the tumor microenvironment, such as selectins and galectins, which are essential for cell adhesion and migration [192]. For example, elevated levels of antigens like Tn, STn, and Lewis antigens on mucins like MUC1, MUC4, and MUC16 have been linked to enhanced tumor metastasis [193]. In colon cancer cells, abnormal *O*-glycosylation of the MUC1 ectodomain leads to increased production of sLeX and sLeA epitopes, which are associated with greater invasive and metastatic potential [136]. Sialyl-Lewis A and sialyl-Lewis X, which serve as ligands for selectins on endothelial cells, support metastasis by promoting the adhesion of tumor cells to the vascular endothelium—a key step in their spread to distant organs [194]. The interaction of TACAs with selectins and galectins highlights the importance of the tumor microenvironment in metastasis, highlighting a potential therapeutic angle by targeting these specific interactions.

##### Altered Glycosylation in Cancer Immune Evasion

Altered glycosylation is instrumental in helping cancer cells avoid recognition and elimination by the immune system. Epigenetic modifications affecting glycan expression on tumor cells can allow them to bypass immune surveillance [136]. Glycan modifications on cancer cells can hinder immune recognition by producing poorly immunogenic surface proteins, enabling immune evasion. One key mechanism involves elevated display of sialic acid on their surface. This terminal glycan can interact with Siglec receptors expressed on various immune cells, including macrophages and natural killer (NK) cells, resulting to the dampening or inhibition of their anti-tumor activity [195]. Glycans and glycan-binding molecules influence nearly every immunological process, highlighting the intricate interplay between the glycome and the immune response in cancer [18]. Galectin-1 contributes to immune evasion by modulating T-cell differentiation, altering dendritic cell interactions, and expanding immunosuppressive regulatory T-cells [196]. High galectin-1 expression in the tumor microenvironment has been associated with enhanced activity of myeloid-derived suppressor cells (MDSCs), which hinder anticancer immunity, leading to poor clinical outcomes [197]. Tumors can exploit these glycan-mediated interactions, creating “glyco-immune checkpoints” to evade immune surveillance. Targeting these checkpoints represents a promising new frontier in cancer therapeutics. The involvement of specific lectins like Siglecs and galectins in immune evasion highlights the potential for developing targeted therapies that disrupt these specific interactions.

##### Aberrant Glycosylation and Resistance to Cancer Therapies

Aberrant glycosylation has been increasingly recognized for its role in the development of resistance to various cancer therapies, including chemotherapy, radiotherapy, and immunotherapy [198]. In colorectal cancer, changes in glycosylation patterns have been associated with chemotherapy resistance by contributing to decreased apoptosis and alterations in drug uptake and metabolism [198]. For instance, decreased fucosylation has been associated with increased resistance to apoptosis in colon cancer cells, while increased *N*-glycan sialylation can block Fas-mediated apoptosis, thereby contributing to resistance [199].

In immunotherapy, aberrant glycosylation’s role in immune evasion can directly impact treatment effectiveness. Prostate cancer cells resistant to enzalutamide show increased sialic acid levels, and blocking sialic acid partially reversed this resistance, demonstrating a direct link [200]. The direct reversal of resistance to a targeted therapy by blocking sialic acid provides a compelling example of the therapeutic potential of targeting aberrant glycosylation.

Abnormal glycosylation is a key characteristic of cancer, significantly influencing tumor development, the spread of cancer cells, immune system avoidance, and resistance to treatment. TACAs, resulting from these altered glycosylation patterns, serve as unique signatures of cancer cells and hold significant potential as both diagnostic biomarkers and therapeutic targets. The field of glycomics has provided valuable insights into the complex interplay between glycans and cancer, revealing promising pathways for the creation of new diagnostic tools and treatment approaches.

### 3.2. Glycan Modifications in Neurodegenerative Disease

In the intricate environment of the nervous system, glycans play indispensable roles in a multitude of processes essential for its development, function, and maintenance. These roles include the precise guidance of neuronal growth during neurodevelopment, the dynamic modulation of synaptic connections underlying synaptic plasticity, the mediation of cell adhesion events crucial for tissue organization, and the regulation of receptor function that governs cellular signaling. Given the extensive involvement of glycosylation in fundamental neuronal processes, it is not surprising that alterations in these glycan structures and their associated enzymatic machinery have been increasingly recognized as critical factors in the etiology and progression of neurodegenerative disorders.

#### 3.2.1. Glycan Modifications in Alzheimer’s Disease

A substantial body of research has now established that Alzheimer’s disease (AD) is characterized by a complex array of alterations in glycan structures within the brain tissue. One of the most consistently observed modifications is the increased presence of bisecting GlcNAc structures in AD brains, particularly within the neurons of the hippocampus and cerebral cortex [127,201]. This specific glycan modification is synthesized by the enzyme *N*-acetylglucosaminyltransferase III (GnT-III), and studies have shown an elevated expression of both GnT-III expression and activity in AD brains [202]. Furthermore, significant alterations in sialylation patterns have been reported in AD. Sialic acids are terminal monosaccharides on glycan chains that are important in cellular recognition and signaling. While some studies indicate a general decrease in overall sialylation levels in AD brain tissue, others suggest that these changes might be specific to certain brain regions or particular glycoproteins [127,203,204]. Changes in fucosylation have also been observed [205,206]. Additionally, investigations have revealed elevated levels of mannosylation and an increase in *N*-glycan truncation in the brain tissue of individuals with AD [207,208]. Beyond these general trends, aberrant *N*-glycosylation of specific proteins implicated in AD pathogenesis has been noted. For instance, tau protein, a key component of neurofibrillary tangles, exhibits *N*-glycosylation at sites that are typically not glycosylated in healthy brains [209]. Zhang et al. (2024) discovered that tau is *N*-glycosylated at asparagine residue N410 by a high-mannose-type glycan exclusively in AD brains, but not in controls [208]. Additionally, a sialylated glycoform at the N359 site was found only in control brains, indicating disease-specific alterations in tau glycosylation associated with AD pathology. Moreover, changes in the metabolism of gangliosides, which are glycolipids carrying sialic acid residues, are increasingly recognized as a contributing factor to amyloid pathology, a hallmark of AD [210].

The alterations in glycan structures observed in AD are not confined to the brain tissue; they are also detectable in various biofluids, including cerebrospinal fluid (CSF), serum, and plasma, offering potential for the development of diagnostic biomarkers. Analyses of CSF from AD patients have revealed distinct *N*-glycan profiles, characterized by a reduction in sialylation and elevation in the presence of bisected GlcNAc structures [211]. Similarly, comprehensive glycomic studies of serum and plasma have demonstrated widespread changes in the *N*-glycosylation of proteins involved in critical biological functions such as immune response, inflammation, and the metabolism of lipoproteins in individuals with AD. Notably, the loss of fucosylation on immunoglobulin G (IgG) in serum has been suggested as a potential diagnostic indicator for AD. Furthermore, specific alterations in the glycosylation pattern of transferrin, a protein present in CSF, have been proposed as a potential biomarker for the disease. Additionally, increased levels of particular glycans bearing bisecting GlcNAc, core fucose, and sialic acid have been identified in the serum of AD patients.

#### 3.2.2. Dysregulated Glycosylation in Parkinson’s Disease

Research employing lectin microarray technology to analyze brain tissue from individuals with Parkinson’s disease has uncovered alterations in a range of glycan structures within both the striatum and the substantia nigra, brain regions critically affected by the disease [34]. A study conducted by Rebelo et al., presents the first comprehensive region-specific analysis of protein *N*-glycosylation in the Parkinsonian brain, revealing significant and distinct glycomic changes in the striatum and substantia nigra [34]. Key findings include increased fucosylation and sialylation, region-dependent alterations in oligomannose and branched glycan structures, and differential regulation of glycosylation-related enzymes and unfolded protein response (UPR) markers. A supporting in vitro study confirmed that inhibition of sialyltransferases leads to mitochondrial dysfunction, altered glycosylation patterns, and activation of ER stress pathways, suggesting that glycosylation changes may contribute to Parkinson’s disease pathogenesis.

Furthermore, an increase in *O*-GlcNAcylation of α-synuclein, the protein whose aggregation is a hallmark of PD, has been detected in PD brain tissue [212,213]. This modification is hypothesized to have a protective function by inhibiting the aggregation of α-synuclein. Glycosylation alterations of other neuroprotective proteins, including TREM2 and the dopamine transporter (DAT), have also been implicated in the pathogenesis of PD [214]. Notably, a decrease in the expression of brain gangliosides, particularly GM1, along with a reduction in the levels of the glycosyltransferases and sialyltransferases responsible for their synthesis, has been reported in PD [215]. Additionally, a decrease in the expression of polysialic acid (PSA), a glycan involved in synaptic plasticity, has been observed in the substantia nigra of individuals with PD [215].

Similar to AD, abnormal protein glycosylation in biofluids such as plasma and CSF has been associated with Parkinson’s disease. Research has shown that patients with Parkinson’s disease exhibit higher levels of serum glycans featuring core fucose, sialic acid, and bisecting GlcNAc [216]. Notably, studies have reported a widespread reduction in the levels of most detected *N*-glycans in the urine of individuals with PD [217]. Additionally, distinct changes in the *N*-glycosylation profiles of key PD-related glycoproteins such as ceruloplasmin and clusterin in serum, and 1-microglobulin/bikunin and uromodulin in urine have been identified, suggesting disease-specific glycosylation alterations that may reflect underlying pathological processes.

#### 3.2.3. The Role of Impaired Glycosylation in Neuroinflammation

Defective glycosylation is gaining recognition as a key factor driving neuroinflammation, a persistent inflammatory response in the brain that is central to the development and progression of neurodegenerative diseases such as AD and PD [34,218]. A central mechanism involves alterations in sialylation patterns, which disrupt the normal inhibitory interactions between neuronal glycans and Siglec receptors on microglia, leading to unchecked microglial activation and a pro-inflammatory state [219]. The increased presence of bisecting GlcNAc on β-site amyloid precursor protein-cleaving enzyme 1 (BACE1), an enzyme crucial in the production of amyloid-beta, has also been linked to chronic inflammation in AD and impaired amyloid-beta clearance [220]. These changes perpetuate microglial activation and the sustained release of neurotoxic cytokines. Similarly, in PD, diminished sialylation of neuronal glycans can promote a pro-inflammatory microglial phenotype [214]. Elevated expression of glycosyltransferases such as *B3GALT2* and *B4GALT1* in the substantia nigra has been linked to enhanced neuroinflammation in PD, while changes in polysialic acid (PSA) levels may further affect inflammatory signaling [215]. Additionally, aberrant glycosylation of disease-associated proteins such as amyloid-β in AD and α-synuclein in PD may alter their interaction with immune cells, thereby influencing downstream inflammatory responses. The dynamic post-translational modification *O*-GlcNAcylation is also emerging as a regulator of neuroinflammation in both diseases, with alterations in its cycling enzymes (OGT and OGA) shown to significantly impact inflammatory pathways [221]. Together, these findings highlight impaired glycosylation as a shared pathological mechanism driving neuroinflammation in AD and PD, offering potential targets for therapeutic intervention.

#### 3.2.4. Role of Impaired Glycosylation in Synaptic Dysfunction

Synaptic dysfunction, characterized by impaired communication between neurons, is one of the earliest pathological events observed in AD, often preceding the more widespread protein aggregation and neuronal loss [222]. Aberrant glycosylation of key synaptic proteins like amyloid precursor protein (APP) and tau can disrupt their normal physiological functions, thereby contributing to the progressive synaptic loss and dysfunction characteristic of AD [223]. Alterations in sialylation can impact synaptic plasticity by regulating the activity of NMDA receptors, which are vital for learning and memory. These changes also affect the function of cell adhesion molecules like NCAM, which are critical for maintaining synaptic structure and stability [224,225]. Furthermore, the decreased levels of HNK-1 glycan observed in AD brains may contribute to both synapse dysfunction and the subsequent loss of synapses [225]. The increased presence of bisecting GlcNAc on BACE1 may indirectly impact synaptic function by promoting the production of neurotoxic amyloid-beta peptides [226]. Additionally, alterations in the composition of gangliosides, which are enriched at synapses, can disrupt normal synaptic transmission [227].

Synaptic dysfunction is also an early and critical characteristic of PD, often preceding the significant loss of dopaminergic neurons in the substantia nigra [228]. Aberrant glycosylation of α-synuclein, the protein that aggregates to form Lewy bodies in PD, can affect its interaction with synaptic vesicles, potentially impairing the release of neurotransmitters at the synapse [229]. Altered glycosylation of the dopamine transporter (DAT), a protein crucial for the reuptake of dopamine from the synaptic cleft, can disrupt dopamine homeostasis, leading to synaptic dysfunction in the dopaminergic pathways that are severely affected in PD [229]. Furthermore, alterations in the metabolism of gangliosides, which are important components of neuronal membranes, may also contribute to synaptic dysfunction in PD [230].

### 3.3. Role of Glycosylation in Cardiovascular Diseases

Glycosylation plays a critical role in cardiovascular diseases by modulating key processes such as inflammation, endothelial function, lipid metabolism, and immune responses [231]. Altered glycosylation patterns, particularly changes in *N*-glycan branching, sialylation, and fucosylation, have been linked to atherosclerosis, hypertension, and myocardial infarction [232,233]. For example, aberrant glycosylation of adhesion molecules like VCAM-1 and ICAM-1 enhances leukocyte recruitment to vascular endothelium, promoting inflammation and plaque formation [234]. Additionally, changes in glycosylation of plasma proteins, such as immunoglobulin G (IgG), are associated with pro-inflammatory profiles that correlate with cardiovascular risk [235]. Glycan modifications on low-density lipoproteins (LDL) also influence their clearance and uptake by macrophages, contributing to foam cell formation and vascular damage [236]. Overall, glycosylation serves as a key regulator of vascular homeostasis and immune balance, and its dysregulation plays a significant role in the pathogenesis of cardiovascular diseases.

A review by Jones and coworkers highlighted the importance of glycosylation in cardiovascular biology [237]. They reported that *O*-GlcNAcylation is especially critical in the heart, modulating development, stress responses, and mitochondrial function. Both deficiency and sustained elevation of *O*-GlcNAc levels impair cardiac structure and performance, as shown in mouse models. Notably, transient increases in *O*-GlcNAc during acute stress, such as ischaemia–reperfusion, offer cardioprotection, while reduced levels exacerbate injury. On the other hand, *N*-glycans are essential for ion channel trafficking and function, with their dysregulation contributing to arrhythmias and contractile dysfunction. Glycosylation also regulates β-adrenergic signaling and GPCR activity, affecting cardiac remodeling. A recent review by Chatham and Patel has expanded the functional landscape of glycosylation in cardiovascular disease by describing its active role in driving pathological cardiac hypertrophy [231]. Beyond its established functions in ion channel regulation and stress response, glycosylation has now been implicated in directly triggering hypertrophic signaling. Altered flux through the hexosamine biosynthetic pathway (HBP), often due to nutrient overload, leads to excessive *O*-GlcNAcylation of transcription factors such as NFAT, NF-κB, and Sp1, thereby promoting the expression of pro-hypertrophic genes [238]. Additionally, disruption of *N*-glycan processing and Golgi function under stress conditions contributes to protein misfolding, ER stress, and maladaptive cardiac remodeling. Golgi fragmentation, a hallmark of glycosylation dysregulation, interferes with membrane trafficking and receptor recycling, further amplifying pathological growth signals. Importantly, specific glycosyltransferases, including members of the MGAT and GALNT families, have been identified as potential molecular regulators of hypertrophic progression. These insights position glycosylation not only as a modulator but also as a mechanistic driver of cardiac hypertrophy, opening new avenues for therapeutic intervention targeting glycoenzymes and nutrient-sensitive glycosylation pathways.

### 3.4. Autoimmune and Inflammatory Disorders

#### 3.4.1. Aberrant Glycosylation in Rheumatoid Arthritis, Lupus, and IBD

The immunological control role played by glycosylation has been an area of interest in the context of autoimmune diseases, among them rheumatoid arthritis (RA), systematic lupus erythematosus (SLE), and inflammatory bowel disease (IBD). Despite advances in clinical understanding of these diseases, a definitive diagnostic and treatment modus operandi remains elusive owing to the multifactorial etiology involving genetics, environmental triggers, microbiome, and immunological disbalance [239,240,241,242,243]. Glycomics, encompassing the extensive study of glycans and the functional role played by them, has shown promise in elucidating the molecular intricacies of these ailments. Glycans are complex carbohydrates attached to lipids and proteins and play pivotal roles in cellular recognition, immunological signaling, and regulation of antibody action [244,245,246]. Maverakis et al. advanced the Altered Glycan Theory of Autoimmunity that stipulated that every autoimmune disease has its own glycan signature defined by site-specific glycosylation changes on proteins and immune cells [10]. They are not controlled by gene expression alone but are modulated by environmental factors and glycan ageing itself.

##### Rheumatoid Arthritis

Rheumatoid arthritis (RA) is an autoimmune disease with chronic synovial inflammation, joint damage, and extra-articular features [247,248,249,250]. Among the multifaceted molecular processes underlying RA, aberrant glycosylation of immunoglobulins and more so IgG has been identified as an indication of disease progression and immune dysregulation. One among the most well-documented glycomic alterations in RA is hypogalactosylation of the Fc arm of IgG on the conserved Asn-297 residue [30]. The resultant structure is one with lower terminal galactose moieties, leading to the formation of G0 glycoforms with an absence of galactose on biantennary *N*-glycan arms [251,252]. Such G0 forms of IgG are pro-inflammatory in nature because these promote increased interaction with Fcγ receptors and activate the complement system through C1q binding and thus sustain immune cell activation and synovial inflammation [253]. Such pro-inflammatory activity correlates with the severity of RA [31,32].

Compared to healthy controls, RA patients presented with characteristic glycosylation patterns, i.e., decreased galactosylation, decreased sialylation, and increased IgG fucosylation [254,255]. Longitudinal investigation identified the extent of IgG hypogalactosylation to correlate with disease activity and to normalize with pregnancy-induced remission, asserting a causative role [256]. Furthermore, the glycosylation is not limited to IgG; glycomic modifications are found in synovial fibroblasts and in T cells in RA and impinge on adhesion, migration, and signaling [257]. Su et al. presented evidence that in the case of RA patients, there existed an inverse correlation between the level of circulating sialylated IgG and rheumatoid factor autoantibodies, suggestive of the level of sialylation as being a suitable serum indicator of RA disease activity and clinical diagnosis [254].

The clinical significance of these findings is considerable. Glycan profiling of IgG in the serum has been suggested to be an attractive non-invasive biomarker to identify disease onset and flare and to monitor treatment [258]. Glycoengineering strategies to reverse anti-inflammatory glycan signatures or abolish pro-inflammatory glycoforms are attractive therapeutic options [259]. Treatments with agents like sialic acid mimetics, glycosyltransferase inhibitors, or glycan-modified Fc monoclonal antibodies could rebalance immunity in RA [260]. Advances in technologies like Multiple Reaction Monitoring (MRM) and LC-MS/MS have made site-specific and high-throughput determination of glycoforms of IgG more feasible and increased the clinical relevance of glycomic biomarkers in rheumatology [261,262,263,264,265,266].

##### Systemic Lupus Erythematosus

Systemic Lupus Erythematosus (SLE) is a prototypic multisystemic autoimmune disease characterized by loss of self-tolerance, generation of pathogenic immune complexes and widespread inflammation in the skin, kidney, joints, and central nervous system. Deranged glycosylation of immunoglobulins with particular emphasis on IgG has increasingly been identified as an important modulator of severity and organ involvement in SLE [267,268]. Sialylation and fucosylation patterns in IgG are profoundly affected in SLE. In ultra-performance LC analyses in more recent times, reduced galactosylation, reduced sialylation, reduced core fucosylation, and elevated bisecting GlcNAc were detected in SLE patient sera compared to healthy controls [269]. Reduced sialylation is known to impair the anti-inflammatory function of IgG and thus promotes tissue damage by way of immune complexes. Likewise, decreased core fucosylation makes the affinity of IgG towards FcγRIIIa greater and increases antibody-dependent cellular cytotoxicity (ADCC), thus aggravating organ damage and particularly lupus nephritis. Lauc et al. reported a reduction in the levels of key sialylated glycans, including FA2G1S1, FA2BG2S1, and FA2G2S2 within total IgG across three patient groups representing diverse ethnic backgrounds [269]. Another study demonstrates that SLE patient anti-histone IgG is under-sialylated compared to patient’s total IgG [270]. Measurement of the extent of non-sialylation of histone reactivity in greater sera can establish itself as an SLE novel serologic marker.

Glycosylation not just modulates effector function but also affects antigen presentation and signaling through the B-cell receptor (BCR) and T cells, both of which are pivotal in lupus pathogenesis [271,272]. Subclass-specific differences further muddy the waters, with unique glycan patterns on the IgG subclasses that can modulate disease manifestation and treatment response [273]. Disease activity scores like the SLE Disease Activity Index (SLEDAI) have been linked to particular glycomic patterns. Thus, decreased galactosylation and sialylation of IgG correlates with flares and renal activity [274,275,276]. Such findings generated interest in glycan-based biomarkers in lupus diagnosis and disease subtype stratification. Mass spectrometry-based glycomics like MRM and glycopeptide-focused LC-MS/MS have made it possible to quantify glycan patterns in the serum with high fidelity in SLE patients. Not just biomarker identification is made easy by this high precision, tracking response longitudinally to therapy is made easy too. Glycoengineering has therapeutic promise to modulate lupus autoimmunity. Strategies like the promotion of increased IgG sialylation or glycan-modified monoclonal antibodies with decreased effector function have not been fully investigated [259].

##### Inflammatory Bowel Disease (IBD)

Crohn’s Disease (CD) and Ulcerative Colitis (UC) are the two main forms of Inflammatory Bowel Disease (IBD), a persistent and recurrent inflammatory disorder that targets the gastrointestinal (GI) tract [277,278]. Defects in glycosylation patterns are consistently found in IBD and are inferred to directly play pivotal roles in disease etiology and progression.

Aberrant glycosylation patterns have been consistently observed in IBD, suggesting their direct involvement in disease etiology and progression. Glycosylation changes affect immunoglobulin function, particularly IgG, which exhibits altered Fc-glycosylation profiles in IBD patients [32]. The defect modulates antibody-dependent cell-mediated cytotoxicity (ADCC) and inflammatory reaction and so enhances gut inflammation. High level agalactosylation or low-level galactosylation of IgG and sialylation of IgG glycans correlate with more aggressive CD and UC [279,280,281]. Based on studies, alterations in patterns of glycosylation in IgG, particularly shifts in the level of galactosylation of IgG, are proposed to act as an analytical tool to reflect IBD disease activity and well as to judge treatment effectiveness. The biggest drawback to managing IBD is the absence of a non-invasive and reliable set of early diagnostic and treatment response biomarkers with which to work. Glycomic profiling presents itself as one area that could offer promise in this context. Thus, galactosylation reduction in IgG correlates with severity in disease and has been put forward as an inflammation biomarker in IBD [280]. Glycomic changes are measurable by taking blood samples and hence are strong contenders to make it into the clinical practice arena. The study of glycomic alterations in IBD unravels the path to novel therapy options. Glycosylation is modulated by enzyme inhibitors, glycan-mimetics or by microbiota-directed therapy and could potentially correct intestinal homeostasis.

#### 3.4.2. Glycan-Mediated Immune Modulation and Inflammation

##### Glycan Roles in Viral, Bacterial, and Parasitic Infections

Attachment and Entry:

Glycans facilitate pathogen adherence and host cell invasion. For viral diseases like HIV, viral envelope glycoprotein glycans act as crucial structures that bind host cellular receptors, thereby facilitating initial attachment and invasion of the virus [16]. The same mechanisms apply in other viral pathogens like influenza, where virus glycoproteins bind host sialylated glycan receptors for viral infection initiation [17]. For bacterial pathogenesis, the pathogen utilizes specific host cell surface-bound glycan structures acting as receptor sites for adherence and colonization. Bacterial surface-bound glycan-binding proteins like lectins and adhesins bind host glycans for attachment and invasion initiation [39,282]. This attachment mediated by glycans is vital in initial bacterial pathogenesis such as that caused by *Helicobacter pylori* [283] and *Streptococcus pneumoniae* [284]. For parasites, the trypanosome species *Trypanosoma cruzi* employ trans-sialidase enzymes that obtain host-derived sialic acids for enhancing invasion of host cells and immune avoidance [285]

Immune Evasion:

Glycans play an important part in pathogen mechanisms for evading host immune response. Viruses, bacteria, and parasites tend to utilize glycan mimicry by decorating themselves with identical or similar host-like glycans, thus hiding their presence and preventing immune detection [286]. HIV-1 uses a glycan shield on its envelope glycoproteins in the form of a dense layer of glycans that shields important epitopes from neutralizing antibodies [287,288]. Bacterial pathogens like *Neisseria meningitidis* and group B Streptococcus also have capsular polysaccharides with host-like glycan structures present on their surface, making them less recognizable by host antibodies and immune cells and thus evading clearance [289,290]. *Trypanosoma cruzi*, a parasite, exploits host glycosylation by adding host-derived sialic acid species on its surface, preventing complement-dependent lysis and immune detection [285].

Pathogenesis:

Glycans have a major impact on disease progression and the severity of infectious disease through multifaceted and varied mechanisms. Aberrant host cell glycosylation during infection plays a critical role in immune responses, receptor signaling, cytokine production, and disease outcome. For example, in viral infection, that of severe influenza displays higher amounts of surface-exposed high-mannose glycans that enhance the binding by innate immune lectins like mannose-binding lectin (MBL) [291]. This augmented contact activates inflammatory cascades leading to cellular damage. Likewise, unique IgG antibody surface glycan signatures during COVID-19 correlate with disease severity and inflammatory response [292,293].

Bacterial pathogens also exploit host glycosylation for the promotion of pathogenicity. For instance, secreted enzymes by Salmonella and vaginosis-causing bacteria decrease host sialylation of glycan, modifying immune signaling and enhancing host susceptibility for secondary infection. Bacterial pathogens also induce elevated fucosylation of the gut glycan, altering microbiome dynamics that influence the severity and outcome of gut infection [294]. Parasitic infection also illustrates the contribution of altered glycosylation in pathogenesis. Parasites such as Leishmania and Toxoplasma gondii functionally alter host glycosylation patterns, with substantial impact on the signaling of immune cells, and they enhance chronic infection states [295]. Furthermore, Toxoplasma-induced alterations in glycans have also been associated with neurological symptoms, further illustrating the wide-ranging and multifaceted pathogenic implications of modulation of the glycans [296].

Overall, glycans and glycan-binding proteins are key factors in the multifaceted interactions between host immunity and pathogens, dictating the whole infectious course from initial infection through chronic disease. Glycan-mediated mechanisms are useful prospects for the development of specific diagnostics, therapeutics, and preventatives of wide-ranging infectious diseases.

Adherence and Colonization:

Bacterial surface proteins called adhesins bind specifically to host tissue glycans, promoting stable colonization. Bacterial lectins that bind mucosal glycans promote colonization of the gastrointestinal tract in gastrointestinal pathogens such as Clostridium difficile and Helicobacter pylori, which is essential for infection [297]. Adherence via glycans also occurs in parasites; for instance, Plasmodium, which infests causing malaria, bind host glycans for attaching and invading erythrocytes, a necessary step for their lifecycle and infection [298].

Invasion and Virulence:

In the case of bacterial pathogens such as Streptococcus pneumoniae and Staphylococcus aureus, the presence of specific capsular glycans improves their virulence and invasiveness by allowing them to penetrate deeper tissue and their spread in the body [299,300]. Glycans presented by fungi such as Sporothrix schenckii are major fungal virulence factors that determine their host immune defense resistivity and their capability of causing disease invasion [301]. Likewise, surface glycans of parasites on such pathogen species as Trypanosoma cruzi play a major role in invasion, host immunomodulation, and colonization in tissues, making them more pathogenic [302].

Immune Evasion/Modulation:

Pathogens utilize glycan architecture to orchestrate host immune responses for immune modulation and long-term infection. Viruses employ viral protein glycosylation for modulation of immune receptor contacts and antibody targeting. For example, SARS-CoV-2 utilizes glycan modifications of spike glycoproteins for modulation of antibody binding and immune responses, and disease severity [303]. Bacteria and parasites also utilize glycan interactions for immune modulation. Parasites like Leishmania hijack the sialylation of Toll-like receptor 4 (TLR4) for the promotion of immune avoidance and survival intracellularly [304]. Bacterial pathogens like group B Streptococcus utilize sialylated glycans for dampening host inflammatory signaling and suppressing effective immune responses [305].

#### 3.4.3. Glycobiology of SARS-CoV-2, Influenza, and HIV

##### Glycobiology of SARS-CoV-2

Glycobiology plays an integral role in understanding virus-host interactions, particularly in viral attachment, entry, immune evasion, and pathogenicity. Below is a comprehensive review of the glycobiology of SARS-CoV-2, influenza, and HIV using the attached articles. The virus responsible for COVID-19, SARS-CoV-2, contains four main structural proteins: spike (S), envelope (E), membrane (M), and nucleocapsid (N). Among these, the S, E, and M proteins undergo extensive glycosylation [306,307]. The S protein contains extensive *N*- and *O*-glycosylation with about 66 *N*-glycosylation sites per trimer, which are essential for receptor binding, immune avoidance, and protein stability. S protein glycosylation involves high-mannose, hybrid, and complex glycans, with the presence of abundant complex-type glycans in key regions such as the receptor-binding domain (RBD) [308]. The spike (S) protein remains a vital target in vaccine development [309]. Glycosylation analysis of recombinant viral spike proteins provides key knowledge towards understanding viral biology and effectively guides vaccine design strategies [28,29,310,311].

The spike glycoprotein interacts with the host receptor, angiotensin-converting enzyme 2 (ACE2) [312]. Host glycosaminoglycans (GAGs), particularly heparan sulfate, also play a pivotal role in attachment by interacting with the viral spike, thereby enhancing binding affinity to ACE2 [313]. Furthermore, viral entry into host cells involves proteolytic cleavage of the S protein by host proteases (e.g., TMPRSS2), a process modulated by glycosylation sites near cleavage regions [312]. Glycosylation on SARS-CoV-2 acts as a shield, protecting epitopes from neutralizing antibodies [314]. The dense glycan “shield” formed by the *N*- and *O*-linked glycans on the spike surface masks protein epitopes, reducing immune recognition and enhancing viral infectivity [315,316]. Additionally, these glycans, being host-derived, decrease the likelihood of eliciting strong immune responses, contributing significantly to viral persistence and pathogenicity. Understanding glycan structure and function on SARS-CoV-2 provides avenues for therapeutic intervention, including monoclonal antibodies targeting specific glycan epitopes or disrupting glycan-receptor interactions. Glycosylation profiles also influence vaccine design, emphasizing the need to mimic natural glycosylation patterns to enhance immunogenicity and effectiveness.

##### Glycobiology of Influenza Viruses

Hemagglutinin (HA) and neuraminidase (NA) are major glycoproteins secreted by Influenza viruses. HA and NA play key roles in the life cycle of the virus, such as host-cell entry and new viral particle release [317]. Glycoproteins of the Influenza virus carry predominantly *N*-linked glycans, such as oligomannose-type and complex-type glycans. Glycans play an important part in influenza virus host specificity and virulence. HA specifically attaches to host cell sialic acid, with human influenza viruses preferring α-2,6-linked sialic acid and birds preferring α-2,3-linked sialic acid [318,319]. Interspecies transmission and the possibility of zoonotic pandemics are determined by such specificity. Glycans on the influenza virus function as a defensive cover, hiding antigenic sites from antibody attachment. Antigenic drift, enabling the virus to avoid existing host immunity and leading to seasonal influenza epidemics and pandemics, results from variations in the patterns of glycosylation [320,321]. Glycan patterns also play a key part in vaccine design, necessitating periodic adjustments in vaccine formulation with regard to antigenic adjustments prompted by partial glycosylation variations.

##### Glycobiology of HIV

HIV envelope glycoprotein gp120 is one of the most extensively glycosylated proteins among viruses, densely covered by host-derived glycans [322]. HIV-1 Env glycosylation consists predominantly of high-mannose glycans (up to 60%), which shield critical epitopes from immune detection and contribute to immune evasion. HIV glycoproteins interact primarily with CD4 receptors and chemokine co-receptors (CCR5 and CXCR4) on host T-cells. Glycans on HIV Env mediate proper protein folding, receptor recognition, and host-cell entry [323,324,325]. Glycan interaction with host lectins facilitates viral entry and transmission between cells, enhancing HIV infectivity. The extensive glycan shield on HIV Env glycoproteins effectively hides critical epitopes from antibodies, complicating the host’s adaptive immune response [322]. This glycan shield continuously evolves through antigenic drift, making neutralizing antibody responses transient and incomplete, thus posing a significant challenge for vaccine development. The dense glycan coverage of HIV has implications for vaccine design and antibody development. Research focuses on eliciting broadly neutralizing antibodies (bnAbs) targeting conserved glycan-dependent epitopes [326,327,328]. HIV vaccine candidates incorporating specific glycosylation patterns aim to expose hidden epitopes and improve immunogenicity.

Table 1 provides an overview of clinically relevant glycan and glycoconjugate biomarkers implicated in cancer, neurodegenerative, and autoimmune diseases.

## 4. Translational Applications of Glycomics in Medicine

### 4.1. Glycan Biomarkers for Disease Diagnosis

Glycan biomarkers have emerged as a powerful and promising tool in disease detection and diagnosis due to their dynamic and disease-specific alterations in glycosylation patterns [114,329]. Their structural modifications often reflect pathological changes in the body, making them valuable indicators of disease progression. Aberrant glycosylation has been strongly linked to various diseases, including cancer, neurodegenerative disorders, diabetes, cardiovascular conditions, and others, where specific glycan signatures serve as early warning signs of disease onset [114,329].

Glycans are highly responsive to environmental and physiological changes, providing insight into disease progression and the impact of external factors such as lifestyle, infection, and inflammation [330]. This dynamic behavior makes glycan biomarkers valuable for monitoring disease dynamics and treatment responses. Moreover, their presence in easily accessible biofluids, such as blood, urine, and saliva, allows for non-invasive or minimally invasive diagnostic approaches [331]. This capability is crucial for early disease detection, enabling timely intervention before significant clinical symptoms arise, ultimately improving patient outcomes and reducing healthcare burdens. In cancer diagnostics, for example, glycan biomarkers have been instrumental in identifying tumor-specific glycan modifications associated with tumor growth, metastasis, and immune evasion [24,114,329]. Similarly, in neurodegenerative diseases like Alzheimer’s, glycan alterations in cerebrospinal fluid and blood provide critical insights into disease mechanisms and progression [332]. In metabolic disorders like diabetes, glycan biomarkers help track glycation changes linked to glucose metabolism and complications [333]. Advanced glycation end products (AGEs), a class of glycans formed through non-enzymatic reactions, play a significant role in diabetes pathogenesis. Jiang et al. demonstrated that elevated skin AGE levels, measured non-invasively via autofluorescence, are associated with increased blood glucose, impaired glucose tolerance, and type 2 diabetes mellitus (T2DM) [334]. AGE accumulation correlated strongly with key metabolic indicators, including BMI, triglycerides, and total cholesterol. Moreover, BMI and other metabolic risk factors such as triglycerides and systolic blood pressure moderated the relationship between AGE levels and glycemic status. Another work by Hu et al. has shown that glycation, through AGE formation, contributes to diabetes by promoting insulin resistance, β-cell dysfunction, and chronic inflammation via RAGE signaling [335]. This accelerates complications like nephropathy and atherosclerosis. AGEs also serve as non-invasive biomarkers for glycemic burden and disease progression.

Beyond diagnostics, glycan biomarkers are increasingly recognized for their role in precision medicine. Their specificity and sensitivity make them ideal candidates for personalized therapeutic strategies, allowing for tailored treatments based on an individual’s unique glycan profile [336,337]. This precision-driven approach promises to enhance treatment efficacy, minimize adverse effects, and optimize patient care [114]. As research advances, integrating glycan biomarkers into routine clinical practice will likely revolutionize disease diagnosis, prognosis, and therapeutic monitoring, making them a focal point in translational medicine and biomedical innovation.

#### 4.1.1. Cancer Glycan Signatures for Early Detection

Cancer significantly alters glycosylation patterns, leading to unique glycan signatures as potential early biomarkers for tumor detection and disease progression [37,330,338,339,340,341,342,343]. Dysregulated glycosylation is a fundamental feature of malignant transformation in cancer cells, contributing to tumor initiation, metastasis, immune evasion, and metabolic reprogramming [37]. The altered expression of glycosyltransferases and glycosidases, which regulate the addition and removal of sugar moieties, leads to structural modifications in glycans that can be leveraged for diagnostic and therapeutic purposes.

A key example of aberrant glycosylation in cancer is the increased sialylation and fucosylation observed in multiple tumor types. Sialylation is frequently upregulated in aggressive cancers, aiding immune evasion by engaging inhibitory Siglec receptors on immune cells and reducing immune surveillance [37]. In breast cancer, particularly basal-like breast cancer (BLBC), aberrant glycosylation of B3GNT5 enhances cancer stem cell properties, increasing invasiveness and resistance to therapy [344]. Similarly, in colorectal cancer, FUT2-mediated α-1,2 fucosylation plays a crucial role in regulating epithelial-mesenchymal transition (EMT) and metastasis by modulating lipoprotein receptor-related protein 1 (LRP1) fucosylation, thereby affecting tumor cell adhesion and migration [345]. Prostate cancer provides another compelling example of glycosylation-driven malignancy, with overexpression of ST6GAL1 catalyzing α2,6 sialylation of *N*-glycans, which promotes tumor growth, invasion, and resistance to apoptosis [346]. A recent review by Eggermont et al. provides a detailed characterization of prostate cancer specific *N*-glycosylation changes beyond sialylation [347]. Prostate cancer is characterized by enhanced branching of complex *N*-glycans, with elevated tri- and tetra-antennary structures driven by upregulation of branching enzymes such as MGAT5 [348,349,350]. These changes correlate with tumor grade, suggesting a role in disease aggressiveness [351]. Core α1,6-fucosylation, mediated by FUT8, is significantly increased in prostate cancer tissue and serum glycoproteins [352,353]. Additionally, the LacdiNAc motif (GalNAcβ1-4GlcNAc) is enriched on PSA and other glycoproteins in prostate cancer tissue, driven by β4-GALNT4 expression, and shows promise as a diagnostic and prognostic biomarker [354]. Moreover, increased expression of the sialyl Lewis X antigen has been detected in prostate cancer tissues and serum, potentially linked to metastatic potential via enhanced GCNT1 activity [351,355]. High-mannose glycans are also elevated in prostate cancer tissues, especially in aggressive and metastatic forms, likely reflecting aberrant Golgi processing or retention of immature glycoforms [350,356]. In hepatocellular carcinoma (HCC), the glycosyltransferase GALNT14 mediates *O*-glycosylation of PHB2, enhancing tumor cell proliferation, migration, and chemotherapy resistance through activation of the insulin-like growth factor 1 receptor (IGF1R) signaling cascade [357]. Additionally, pancreatic ductal adenocarcinoma (PDAC) cells exhibit increased glycosylation of malate dehydrogenase 1 (MDH1), facilitating glutamine metabolism and promoting tumor survival under metabolic stress conditions [358].

Given their functional significance, glycan structures are being leveraged for diagnostic and therapeutic purposes. Clinical biomarkers like carcinoembryonic antigen (CEA), alpha-fetoprotein (AFP), and prostate-specific antigen (PSA) rely on altered glycosylation patterns [37]. More recently, the *N*-glycosylation of immune checkpoint molecules such as PD-L1 has been recognized for its role in stabilizing the protein and promoting immune evasion, making glycosylated PD-L1 a promising biomarker for immunotherapy response [359].

Advancements in analytical techniques have significantly enhanced glycan detection and characterization. Mass spectrometry (MS), especially tandem MS (MS/MS), is central to glycan analysis. Fragmentation techniques like collision-induced dissociation (CID), infrared multiphoton dissociation (IRMPD), electron dissociation (ExD), and ultraviolet photodissociation (UVPD) provide structural insights, including glycosidic linkages [360]. Enzymatic digestion with exoglycosidases complements MS/MS by confirming monosaccharide sequence and linkage. These enzymes selectively cleave terminal monosaccharide residues, allowing for a stepwise determination of glycan structures when combined with separation techniques like high-performance liquid chromatography (HPLC) and capillary electrophoresis (CE). Another powerful tool for structural elucidation is nuclear magnetic resonance (NMR) spectroscopy, particularly when integrated with LC-MS/MS. Although NMR directly confirms glycan branching and linkages, its sensitivity limitations make it more suitable for analyzing abundant glycans [360]. Additionally, glycan microarrays offer an alternative approach by employing metadata-assisted glycan sequencing (MAGS), where glycan-binding proteins help identify biologically relevant motifs.

Glycan quantitation methods vary depending on the desired precision level and the sample’s nature. Label-free quantification is commonly used in glycomic studies, leveraging nanoLC-TOF-MS to detect glycans without chemical modifications. In this approach, glycopeptide signal intensities are normalized to either a specific glycoform or the total glycan pool, making it suitable for *N*- and *O*-glycopeptides. For more precise relative quantitation, stable isotope labeling techniques, including isotopic permethylation, reductive amination, glycan reduction, and ^18^O incorporation via PNGase F digestion, provide enhanced accuracy by comparing label ratios. Targeted techniques such as multiple reaction monitoring (MRM) and parallel reaction monitoring (PRM) increase sensitivity and specificity in targeted glycopeptide quantification [361,362,363]. Another crucial aspect of glycan quantitation is glycosylation site occupancy analysis, which assesses the extent of glycosylation at specific protein sites. This method typically involves enzymatic deglycosylation using PNGase F or Endo H, followed by MRM-based quantification. Such analyses are particularly valuable in studying glycosylation-related diseases and quality control for monoclonal antibody-based therapeutics. Together, these techniques provide comprehensive insights into glycan structures and their biological significance, advancing our understanding of glycosylation in health and disease.

#### 4.1.2. Glycoproteins as Non-Invasive Biomarkers in Neurodegeneration

Glycoproteins from non-invasive biofluids such as blood serum, plasma, urine, and saliva are valuable biomarkers for several diseases, including neurodegenerative diseases (ND) such as AD and PD, aiding early detection and monitoring. Key biomarkers include Tau, α-synuclein, lysosomal enzymes, immunoglobulin G (IgG), amyloid-β (Aβ) peptides, glial fibrillary acidic protein (GFAP), and Neurofilament Light Chain (NfL). These glycoproteins are abundant in non-invasive biofluids, making them amenable to analysis without invasive procedures. Their disease-specific glycosylation changes reflect roles in neural function, cell adhesion, and communication. Advances in mass spectrometry and glycomics now enable more sensitive detection, supporting earlier and more accurate ND diagnosis and treatment.

The amyloid precursor protein (APP) is a glycosylated transmembrane protein predominantly expressed in neurons but also found in vascular and blood systems. BACE1 and γ-secretase generate Aβ peptides, such as Aβ40 and Aβ42 [364]. These peptides, particularly Aβ42, aggregate into oligomers and plaques, central pathological hallmarks of AD and patients with mild cognitive impairment (MCI) [365]. Dysregulation of the Aβ pathway, including increased production or impaired clearance of Aβ, contributes to neurodegenerative cascades that precede tau pathology, synaptic failure, and cognitive decline in AD [366]. Genetic evidence, such as APP, PSEN1, and PSEN2 gene mutations, underscores Aβ dyshomeostasis’s role in early-onset AD [366,367]. At the same time, risk alleles like APOE ε4 exacerbate Aβ accumulation in late-onset AD [368]. These findings have propelled the development of Aβ-targeting therapies. Several of these, including monoclonal antibodies like aducanumab and lecanemab, have gained FDA approval as disease-modifying treatments for early-stage AD or MCI by reducing amyloid plaque burden [369,370]. Pathological glycosylation patterns have also been linked with key pathological proteins, such as tau in AD. Increased *N*-glycosylated tau and dysregulated levels of *O*-GlcNAcylation have been linked with the disease, impacting protein aggregation and neurotoxicity [371,372,373].

Altered glycosylation patterns have been linked to PD pathology, particularly in α-synuclein and IgG. *O*-GlcNAcylation of α-synuclein at specific residues such as Tyr72 and Ser87 has been shown to inhibit its aggregation and toxicity, highlighting its role in disease modulation [374,375]. Furthermore, profiling of plasma *N*-glycans on IgG in patients with PD showed notable alterations, such as decreased sialylation and increased fucosylation on tri-antennary glycans bearing α2,3-linked sialic acids [376].

GFAP and NfL are critical biomarkers of neurodegenerative diseases, each reflecting distinct aspects of disease pathology. GFAP, a well-established marker of astrocyte activation and neuroinflammation, is notably elevated in AD. This increase is primarily driven by its link to Aβ plaque accumulation and reactive gliosis [377,378]. Clinical studies have shown that GFAP levels can effectively differentiate AD from MCI, with a diagnostic cut-off value of 46.05 pg/mL and strong disease specificity [377,379]. On the other hand, NfL is a marker of axonal injury, making it a more general indicator of neurodegeneration. Elevated NfL levels are observed not only in AD but also in other conditions, such as frontotemporal dementia (FTD) and multiple sclerosis, reflecting its broader diagnostic range [377,379,380]. While GFAP and NfL levels are elevated in the early stages of AD and MCI compared to healthy individuals, GFAP demonstrates superior diagnostic performance [377]. Importantly, longitudinal studies have shown that increased levels of GFAP and NfL can predict the onset of dementia as early as 15 years before clinical symptoms appear [379,380]. GFAP strongly correlates with cognitive decline, highlighting its potential as a prognostic marker [379,380]. GFAP and NfL offer enhanced diagnostic sensitivity and specificity when used together, underscoring their complementary value in early disease detection and monitoring. Despite these shared benefits, the distinct pathological associations of each biomarker, GFAP with Aβ-driven astrocytosis and NfL with general neuronal injury demonstrate their unique contributions to our understanding of neurodegenerative processes [378,379].

### 4.2. Therapeutic Targeting of Glycans

Glycoengineering, the deliberate modification of glycosylation patterns on therapeutic molecules, is rapidly emerging as a powerful tool for enhancing the performance and precision of modern medicines. By fine-tuning glycans, scientists can improve critical drug attributes such as target specificity, pharmacokinetics, immunogenicity, and therapeutic efficacy. One of its most established applications is in monoclonal antibody (mAb) development. Beyond mAbs, glycoengineering also offers innovative strategies for improving the stability and bioavailability of biologics. Techniques such as glycan masking, where immunogenic sites on a therapeutic protein are shielded using polymers like polyethylene glycol (PEG), help reduce off-target interactions, lower immunogenicity, and extend the drug’s half-life in circulation [381]. These approaches contribute to safer, more effective treatment regimens, particularly in chronic and immune-sensitive conditions. Glycoengineering also plays a growing role in vaccine development, where synthetic glycans mimic pathogen-associated carbohydrates to elicit strong, targeted immune responses against infections or tumors. Additionally, the emergence of glycosite-specific antibody-drug conjugates showcases how precise glycan modifications can be leveraged to control drug-to-antibody ratios, improving therapeutic potency while minimizing systemic toxicity. As glycobiology and genetic engineering tools advance, glycoengineering is redefining therapeutic design and delivery, bridging molecular innovation with clinical application, and driving progress toward personalized, patient-centered medicine.

#### 4.2.1. Glycoengineered Monoclonal Antibodies

mAbs have transformed modern medicine with targeted therapies for cancer, autoimmune, and infectious diseases. However, their efficacy depends not just on antigen binding, but also on Fc-region glycosylation, particularly the structure of *N*-linked glycans, which critically influences their therapeutic performance. These glycan moieties modulate key effector functions such as antibody-dependent cellular cytotoxicity (ADCC), complement-dependent cytotoxicity (CDC), and serum half-life and can also influence immunogenicity. As a result, glycoengineering, the deliberate modification of glycan structures, has emerged as a powerful tool for optimizing antibody-based therapeutics [382,383].

The Fc region of IgG antibodies is typically glycosylated at Asn297, and this single glycosylation site significantly impacts interactions with Fc gamma receptors (FcγRs) on immune effector cells. Antibodies lacking core fucose exhibit markedly enhanced binding to FcγRIIIa on natural killer (NK) cells, leading to increased ADCC. This finding has driven the development of afucosylated antibodies, which are now part of several approved therapies [384,385]. Additionally, increased galactosylation can enhance CDC by promoting better C1q binding, while terminal sialylation may shift antibodies toward anti-inflammatory functions, as observed in intravenous immunoglobulin (IVIG) preparations [386].

**Figure 3:**
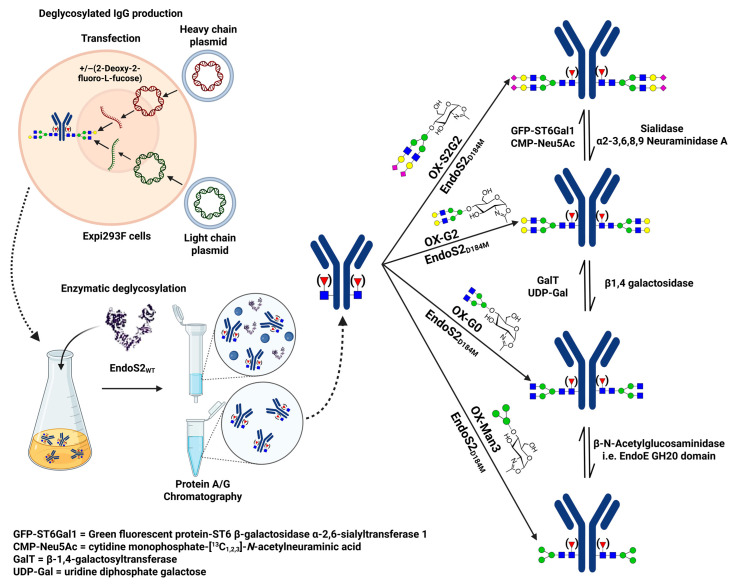
Chemoenzymatic Glycoengineering Strategies for IgG Remodeling. This schematic outlines a versatile workflow for the site-specific remodeling of Fc *N*-glycans on IgGs using chemoenzymatic approaches. Recombinant IgGs bearing heterogeneous complex-type *N*-glycans are expressed in Expi293F cells transfected with heavy and light chain plasmids. These antibodies can be produced in the presence or absence of the fucosylation inhibitor 2-deoxy-2-fluoro-L-fucose to control core fucosylation. Following purification by Protein A/G chromatography, native glycans are selectively removed from the Fc region using EndoS2 WT, leaving a single *N*-acetylglucosamine (GlcNAc) residue, with or without core fucose. The deglycosylated IgGs are then remodeled using the mutant glycosynthase EndoS2 D184M and chemically activated glycans to generate homogeneous glycoforms. Additional enzymatic treatments enable further fine-tuning of the glycan structures. This strategy facilitates precise control over IgG glycosylation for functional and therapeutic studies. Reproduced with permission from García-Alija et al. [396].

Glycoengineering is achieved through several complementary approaches. Cell line engineering remains the most common strategy, particularly in Chinese hamster ovary (CHO) cells. Genetic modification of these cells to knock out fucosyltransferase (FUT8), or overexpress glycosyltransferases such as β1,4-galactosyltransferase or α2,6-sialyltransferase, enables the production of antibodies with tailored glycan profiles [387,388]. Alternatively, in vitro enzymatic remodeling using glycosidases and glycosyltransferases allows precise, post-expression editing of glycan structures [389]. Another promising approach is metabolic glycoengineering, where precursor sugars are supplemented during cell culture to influence glycan biosynthesis [390]. Figure 3 shows a schematic representation of IgG glycoengineering using a chemoenzymatic approach. Several glycoengineered mAbs have translated into clinical success. Obinutuzumab (Gazyva^®^), an anti-CD20 antibody for chronic lymphocytic leukemia and follicular lymphoma, was glycoengineered for afucosylation to enhance ADCC activity and improve efficacy compared to rituximab [391]. Similarly, mogamulizumab, an anti-CCR4 mAb approved in Japan for treating adult T-cell leukemia/lymphoma, was engineered with low-fucose glycans to potentiate immune effector functions [392]. These examples highlight how subtle modifications in glycan architecture can lead to significant therapeutic gains.

A notable case study in glycoengineering is the development of trastuzumab (Herceptin^®^) and its next-generation derivative margetuximab. Trastuzumab is a humanized IgG1 mAb targeting the HER2 receptor, widely used in the treatment of HER2-positive breast and gastric cancers. While trastuzumab inhibits HER2 signaling and mediates ADCC, its clinical efficacy can be affected by patient polymorphisms in FcγRIIIa. Particularly, individuals with the low-affinity FcγRIIIa-158F variant tend to exhibit a diminished response to trastuzumab due to weaker immune activation [393]. To address this, margetuximab (Margenza^®^) was developed as a glycoengineered Fc-optimized version of trastuzumab. It incorporates five amino acid substitutions in the Fc region to enhance affinity for activating FcγRIIIa and reduce binding to the inhibitory FcγRIIb, thereby improving immune effector recruitment, especially in patients with suboptimal FcγR genotypes [394]. Notably, margetuximab retains the same Fab region as trastuzumab, preserving HER2 specificity, while offering enhanced ADCC through Fc modifications. In parallel, afucosylated trastuzumab variants produced in engineered CHO cells have demonstrated up to 50-fold increased ADCC activity in vitro, further emphasizing the therapeutic potential of Fc glycan modulation [385]. These innovations illustrate the growing role of glycoengineering in overcoming biological variability and customizing antibody function for improved clinical response. Ongoing research is exploring more refined glycan modifications such as bisected GlcNAc, high-mannose structures, and Fab glycosylation, all of which hold potential to further fine-tune mAb behavior [395]. Integration of glycomics, glycoproteomics, and artificial intelligence is paving the way for next-generation antibodies with predictable, programmable glycosylation patterns, expanding the capabilities for personalized and precision medicine.

#### 4.2.2. Glycan-Based Vaccines and Immunotherapy Strategies

Glycans have gained increased attention as crucial modulators of immune responses [330,338,339,340,341,342,343]. The structural diversity and disease-specific expression of glycans make them promising targets for vaccines and immunotherapies. Glycan-based vaccines stimulate immune responses against carbohydrate antigens on pathogens or tumor cells, offering an alternative to protein-based vaccines. By targeting tumor-associated carbohydrate antigens (TACAs) and microbial glycoconjugates, this approach advances infectious disease prevention and cancer immunotherapy, overcoming challenges of immune tolerance and specificity [397].

A key application of glycan-based immunology is in carbohydrate-conjugate vaccines for encapsulated bacteria like *Streptococcus pneumoniae*, *Neisseria meningitidis*, and *Haemophilus influenzae* type b (Hib). Their polysaccharide capsules are weakly immunogenic alone but, when conjugated to carrier proteins (e.g., diphtheria or tetanus toxoids), trigger strong T-cell dependent responses with immune memory and affinity maturation [398]. For example, the Hib conjugate vaccine, introduced in the late 1980s, dramatically reduced the incidence of pediatric meningitis and other Hib-related infections worldwide. Similarly, the 13-valent pneumococcal conjugate vaccine (PCV13), which targets 13 different *S. pneumoniae* serotypes, has significantly lowered rates of invasive pneumococcal disease in children and adults [399]. These successes substantiate the immunological potential of glycan antigens when properly presented and provide a foundation for extending glycan-based vaccine design to more complex targets such as viruses and eukaryotic parasites.

Cancer cells often show abnormal glycosylation, producing TACAs. These glycan antigens have become promising targets for cancer vaccines and immunotherapies. Though non-protein, TACAs can be made immunogenic by conjugation to carrier proteins or multivalent formulations. Theratope, an STn-KLH vaccine, showed early promise in metastatic breast cancer but failed to demonstrate broad efficacy in phase III trials [400,401]. Nevertheless, this effort laid critical groundwork for the optimization of TACA-based immunotherapies. More recently, cancer vaccine research has focused on multivalent glycan presentations and the use of nanoparticle delivery systems to enhance immunogenicity [402]. A preclinical study on a globo H-KLH vaccine demonstrated both safety and antibody induction in prostate cancer patients, supporting further clinical development [403]. Similarly, efforts to target GM2 gangliosides have shown promise in melanoma, leveraging glycan-specific monoclonal antibodies and conjugate vaccine platforms [404].

Siglec inhibitors are emerging as promising cancer immunotherapeutics by blocking the interaction between tumor-associated sialoglycans and inhibitory Siglec receptors on immune cells [199,405,406]. This disruption restores anti-tumor immunity by reversing Siglec-mediated immune suppression, particularly of natural killer (NK) cells and macrophages. Preclinical studies demonstrate that targeting Siglecs, especially Siglec 7, 9, and 15, enhances immune cell activation and improves response to existing therapies, positioning Siglec blockade as a valuable strategy in cancer immunotherapy [407,408].

Modern glycan-based immunotherapy leverages advanced strategies to enhance immune targeting, including nanoparticle delivery systems and glycan-engineered immune cell therapies. Multivalent glycan displays on gold nanoparticles and biodegradable scaffolds improve antigen presentation and tumor targeting, with glycan-based vaccines targeting TACAs like Globo-H and GD2 now in clinical trials for breast, ovarian, and prostate cancers [409]. These approaches are increasingly combined with immune checkpoint inhibitors, as glycosylation directly regulates PD-L1 stability and Siglec-mediated immunosuppression [410]. CAR-T cells targeting glycan epitopes such as sialyl-Tn and GD2 show clinical promise, while glycan-modified nanoparticles enhance drug delivery and cytotoxic responses in melanoma and pancreatic cancer models [409,410]. In infectious diseases, glycan-conjugate vaccines for pathogens like Haemophilus influenzae demonstrate proven efficacy, with similar strategies adapted for cancer-specific antigens [409,410]. For SARS-CoV-2, Huang et al. (2022) developed a glycoengineered spike protein vaccine by enzymatically trimming all *N*-glycans to a single GlcNAc residue, generating the mono-GlcNAc-decorated S protein (SMG) [315]. Immunization with SMG in mice elicited stronger humoral and cellular immune responses than the fully glycosylated form, including higher neutralizing antibody titers, enhanced T-cell activation, and broader B-cell diversity. SMG conferred superior protection against multiple SARS-CoV-2 variants in animal models and enabled the isolation of a broadly neutralizing antibody. Glycan-based vaccines and immunotherapies are an emerging and promising area in biomedical research, showing proven success in bacterial infections and expanding potential in cancer and viral diseases. Although challenges like low immunogenicity and self-tolerance exist, advances in glycan design, delivery systems, and adjuvants are driving progress. Integrating glycomics, immunology, and synthetic chemistry offers new opportunities for developing effective treatments and preventive strategies for complex diseases.

### 4.3. Glycoengineering for Precision Medicine

Glycoengineering has demonstrated valuable applications in precision medicine, enabling the design of therapies tailored to individual patients by modifying the glycosylation patterns of therapeutic proteins. In precision medicine, glycoengineering optimizes glycoforms of antibodies and other biologics, enhancing their ability to target disease-specific cellular markers while minimizing off-target effects. For example, engineered CAR T-cell therapies leverage glycomic insights to improve tumor specificity and patient response rates in leukemia-related cancers. Unlike genomic data alone, glycan profiles integrate genetic and environmental factors, offering a dynamic layer of personalization for treatment strategies. This approach aligns with the broader goals of precision medicine by addressing interpatient variability at the molecular level, enabling therapies that account for individual differences in disease mechanisms and drug responses [411].

#### 4.3.1. Personalized Glycoprofiling in Drug Response and Efficacy

Glycoprofiling is increasingly recognized as essential in personalized medicine, playing a significant role in drug absorption, distribution, metabolism, and excretion (ADME) [412]. Glycosylation affects drug stability, modulates receptor binding, and influences clearance pathways. Specific glycosylation patterns can alter pharmacokinetics by affecting proteolytic resistance and receptor interactions in therapeutic proteins, including mAbs [412]. Sialic acid-terminated glycans contribute to prolonged drug half-life by decreasing hepatic clearance, enhancing therapeutic efficacy [412,413]. This principle is exemplified by darbepoetin alfa, a hyperglycosylated erythropoiesis-stimulating agent, which exhibits an extended circulation time primarily attributed to its optimized glycosylation profile [414,415]. Beyond improving pharmacokinetics, glycosylation has also been strategically utilized in targeted cancer therapies to enhance drug specificity and reduce systemic toxicity. One notable example is the glycosylated prodrug Gal-DOX, which incorporates a galactose moiety to facilitate selective binding to the asialoglycoprotein receptor 1 (ASGPR1), commonly overexpressed on specific tumor cells. This targeted approach allows for preferential accumulation in malignant tissues while minimizing uptake by normal cells, thereby improving therapeutic outcomes and minimizing adverse effects [416,417]. In chemotherapy and immunotherapy, glycoprofiling has been instrumental in predicting drug efficacy and toxicity. P-glycoprotein, an efflux transporter influenced by glycosylation, plays a critical role in multidrug resistance by limiting intracellular drug accumulation. Inhibitors targeting P-glycoprotein have shown variable success in overcoming chemoresistance [418]. Moreover, glycoengineering of antibodies for immunotherapy has optimized their binding to Fcγ receptors, enhancing antibody-dependent cellular cytotoxicity. For example, afucosylated IgG1 antibodies have demonstrated improved tumor clearance by boosting immune cell activation [419]. These advancements emphasize the potential of personalized glycoprofiling to tailor therapies based on individual glycan profiles, thereby maximizing therapeutic efficacy while minimizing adverse effects.

#### 4.3.2. Glycobiology of Biopharmaceuticals and Gene Therapy

Glycosylation is essential in the development and optimization of biopharmaceuticals and gene therapies by significantly influencing protein stability, immunogenicity, solubility, and targeting efficiency [420]. It is a critical quality attribute that impacts the safety, efficacy, and pharmacological properties of therapeutic agents [420]. More than 50% of approved protein therapeutics, including mAbs, are glycoproteins, and their glycosylation patterns are known to affect pharmacokinetics, receptor interactions, and serum half-life [412,413]. For example, the presence or absence of terminal sialic acids, fucose residues, or high-mannose structures can alter ADCC, clearance rates, and receptor-binding affinities [419]. To reduce immunogenicity and prolong circulation time, glycan shielding techniques such as PEGylation (attachment of polyethylene glycol chains) are employed to mask immunogenic epitopes and enhance biostability [421]. In gene therapy, glycosylation is also gaining attention as a modulator of vector performance. Adeno-associated virus (AAV) vectors, particularly AAV9, exhibit glycosylation-dependent tissue tropism and transduction efficiency. Recent proteomic and glycomic analyses have uncovered the presence of low-abundance *N*- and *O*-linked glycans on AAV9 capsid proteins, which appear to modulate receptor binding and contribute to immune evasion mechanisms [422]. These advances in glycoengineering have enabled the deliberate modification of viral vector glycosylation to optimize delivery and reduce off-target effects. Altering the levels of sialylation or fucosylation on AAV serotypes has shown promise in improving tissue-specific uptake and minimizing neutralizing antibody responses, thereby enhancing therapeutic outcomes [422]. Furthermore, glycan-targeting strategies, such as conjugating drug delivery systems to ligands that recognize tumor-specific glycans, offer a powerful approach to increase precision in targeting cancer cells while sparing healthy tissue [114]. These developments emphasize glycosylation’s importance as a cornerstone in biopharmaceutical innovation. Rigorous glycan profiling and control are essential throughout the drug development pipeline, from early discovery to large-scale manufacturing, to ensure consistency, therapeutic efficacy, and patient safety.

## 5. Challenges and Future Perspectives in Glycomics and Disease Research

### 5.1. Challenges in Glycan Analysis and Interpretation: Structural Complexity and Isomeric Diversity

Glycan structural complexity poses significant challenges for identification, largely due to isomerism arising from variations in branching, linkage, and stereochemistry. Although glycans may share the same composition, their structures can differ greatly. This diversity results from biosynthesis driven by a dynamic, cell-specific network of glycosyltransferases and glycosidases, rather than direct genetic coding [36]. As a result, distinguishing glycan isomers is a critical task in glycomics. Glycan structures may have identical monosaccharide compositions but differ in glycosidic linkages, producing isomers with the same mass yet distinct biological functions and interactions [423,424]. Advanced analytical techniques like ion mobility spectrometry (IMS), capillary electrophoresis (CE), liquid chromatography (LC), along with mass spectrometry (MS), have proven essential for resolving and identifying these complex structures [425,426,427,428]. Specialized separation columns, including porous graphitized carbon (PGC) and mesoporous graphitized carbon (MGC), have been employed to improve isomeric resolution in MS-based workflows [429,430,431,432].

The biological relevance of glycan isomerism is further emphasized by its impact on molecular recognition. Glycan-binding proteins, such as lectins, often discriminate between specific isomeric forms, influencing immune responses and cell signaling pathways [15,433]. Therefore, the ability to resolve and annotate these subtle differences is crucial for understanding the functional roles of glycans, especially in the context of modifications like sialylation and fucosylation [434]. However, the field continues to face limitations due to a lack of standardized glycan libraries, well-characterized reference materials, and comprehensive structural databases. These gaps hinder accurate quantification and comparative studies across laboratories. Addressing these limitations through improvements in separation technologies, data analysis tools, and standardization protocols will significantly enhance our capacity to interpret glycan function and complexity in health and disease.

Beyond experimental and analytical complexities, glycomics research is increasingly hindered by challenges in data interpretation, integration, and reproducibility. The vast structural diversity and branching complexity of glycans demand sophisticated computational frameworks for accurate annotation, comparison, and biological contextualization. Several computational tools have been developed to support glycomics analysis by facilitating data processing, visualization, and structural interpretation. Tools such as MultiGlycan [435], SimGlycan [436], nQuant [437], GlyReSoft [438], Glycolyzer [439], and GlyCombo [440] are widely used for glycan structure annotation and quantification. These platforms enhance the reliability and reproducibility of glycomics data by enabling accurate peak detection, spectral matching, and database-assisted identification, often serving as foundational components in both discovery and targeted glycomics pipelines. Resources such as GlyTouCan, the international glycan structure repository, and UniCarb-DB, a curated MS/MS glycomic spectral database, have become foundational for glycan structure registration, metadata standardization, and data sharing across platforms [441,442]. However, the absence of unified nomenclature systems, inconsistent metadata reporting, and fragmented data formats continues to impede cross-study comparisons and large-scale meta-analyses. To address these limitations, initiatives like the GlycoBioinformatics Consortium and the Glycome Informatics Consortium (GLIC) have prioritized the development of universal data formats, glycan ontologies, and interoperable software standards [442]. These efforts are essential for integrating glycomics with other omics layers, such as transcriptomics and proteomics, and for enhancing the translation utility of glycan-based biomarkers in disease diagnostics and therapeutic design. Despite notable progress, glycoinformatics pipelines remain underdeveloped relative to those in genomics and proteomics. The lack of robust machine learning tools, automated structure annotation algorithms, and scalable data repositories highlights the need for sustained investment in glycoinformatics infrastructure. Continued community-wide standardization and collaborative tool development will be pivotal in unlocking the full potential of glycomics in systems biology and precision medicine.

#### Standardization and Reproducibility Issues

A major barrier in glycomics research is the lack of standardization, which directly affects data reproducibility and comparability across studies. Glycan analysis typically involves multiple complex steps, including derivatization, separation, detection, and data interpretation, all of which can introduce variability and influence results [443,444,445]. Differences in enzyme specificity or activity further contribute to inconsistent glycan profiles, particularly when analyzing diverse biological samples [446].

**Figure 4:**
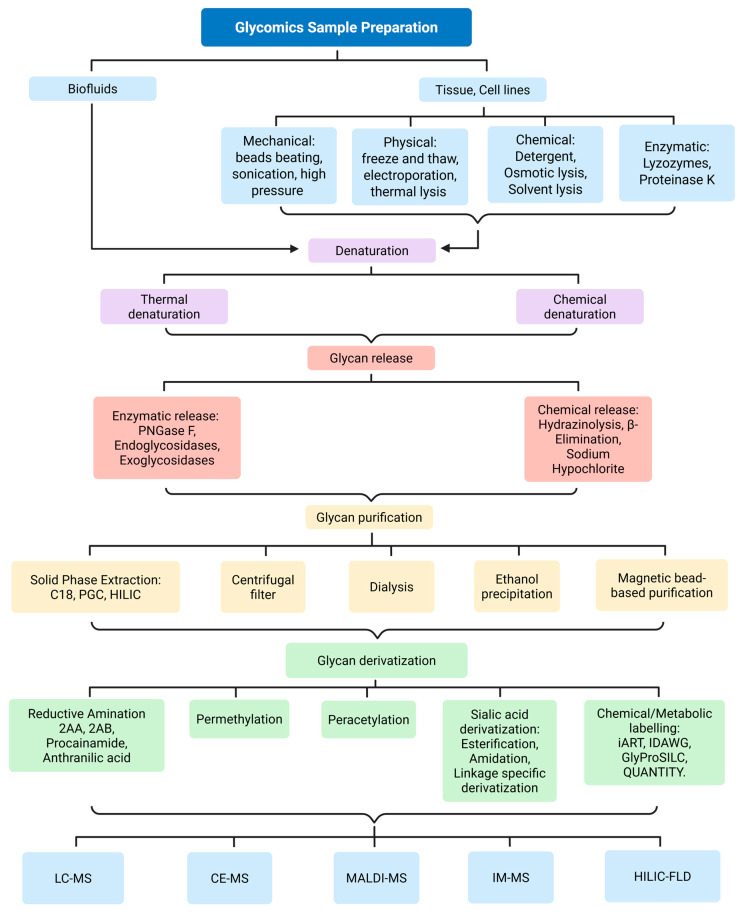
The workflow representation of glycan analysis illustrating various techniques employed at different stages of glycomics workflow, including glycan release, purification, derivatization, and detection. This figure highlights the diversity of physical, chemical, and enzymatic strategies used depending on sample type and analytical goals, as well as a range of mass spectrometry and fluorescence-based detection methods. This broad array of methods, while offering flexibility, contributes to challenges in standardization and reproducibility across laboratories and studies, necessitating the development of harmonized protocols for comparative glycomics.

One of the key issues is the absence of universally accepted protocols. Many researchers rely on in-house methods tailored for specific sample types or structural targets, making it difficult to harmonize findings across different laboratories or large datasets. This variability is illustrated by the wide range of analytical workflows currently used in glycomics analysis (Figure 4). While database-assisted tools are frequently used in glycomics, the field still lacks fully automated and standardized software solutions for reliable glycan and isomeric identification and quantification. This limitation, combined with variability introduced by minor changes in analytical conditions, such as sample denaturation methods, glycan release, derivatization techniques, cleanup methods, and separation conditions, can lead to inconsistencies in glycan profiles across studies.

To improve reproducibility and cross-study comparability, there is a critical need for standardized analytical workflows, robust bioinformatics tools, and comprehensive, open-access glycan databases. The development of these tools is critical for advancing the clinical utility of glycomics, particularly in biomarker discovery and systems biology applications.

### 5.2. Emerging Trends in Glycomics

Glycomics is rapidly evolving, with advances like single-cell and spatial glycoprofiling enabling cell- and tissue-specific analysis of glycan heterogeneity in complex diseases. Integrating glycomics with proteomics, metabolomics, and transcriptomics offers system-level insights into glycan function, disease mechanisms, and biomarker discovery.

#### 5.2.1. Single-Cell Glycomics and Spatial Glycoprofiling

The emergence of single-cell glycomics and spatial glycoprofiling represents a paradigm shift in glycobiology, addressing long-standing limitations in conventional bulk glycan analysis [447,448]. Traditional glycomic techniques average glycan signals across heterogeneous cell populations, often obscuring rare or cell-type-specific glycosylation patterns that are critical in understanding complex biological processes and disease states. Recent advances now allow for glycan profiling at the single-cell level, using technologies such as microfluidic platforms, single-cell mass spectrometry, and lectin microarrays [449,450]. These tools enable the resolution of cell-specific glycan signatures, uncovering subtle but biologically significant differences in glycosylation that are linked to cell differentiation, immune modulation, and disease progression, particularly in cancers, autoimmune disorders, and neurodegenerative diseases [451,452,453].

Spatial glycoprofiling complements this by offering insights into where specific glycans are expressed within tissues. Techniques like mass spectrometry imaging (MSI), fluorescent lectin imaging, and glycan-targeted probes now permit the high-resolution visualization of glycan distribution in situ [454,455]. This spatial context is crucial in identifying tumor margins, immune cell infiltration zones, or regions of inflammation, where glycan expression often varies drastically and informs cellular interaction, tissue remodeling, and drug response. In oncology, spatial glycomics has revealed distinct glycan patterns associated with tumor progression, immune evasion, and metastasis, including alterations in sialylation, fucosylation, and *O*-linked glycan branching [456,457]. In the central nervous system, spatial glycoprofiling has been applied to understand glycan-related mechanisms in synaptic function, neuronal communication, and neuroinflammation, providing clues into diseases like AD and PD [34,458].

Despite their transformative potential, these approaches face technical challenges. Single-cell glycomics often suffer from low glycan abundance, limited sensitivity, and complex data interpretation, while spatial methods are constrained by instrument resolution, sample preparation variability, and standardization issues. Integration with transcriptomic or proteomic data at single-cell resolution also remains technically demanding but is a promising frontier for systems-level glycobiology. Going forward, the development of more sensitive detection technologies, miniaturized analytical platforms, and integrative bioinformatics pipelines will be crucial for translating these techniques into routine research and clinical applications. As the field matures, single-cell and spatial glycomics are expected to provide unprecedented insights into the spatiotemporal dynamics of glycosylation, enabling personalized diagnostics, therapeutic stratification, and biomarker discovery in precision medicine.

#### 5.2.2. Integrative Multi-Omics Approaches for Unraveling Disease Mechanisms

The combination of glycomics with proteomics and metabolomics is important for decoding complex biological systems and uncovering new insight of disease mechanisms [459,460]. The integrative analysis of different omics highlights correlative perception. Proteomics represents alteration of protein expression and modification, metabolomics highlights metabolic changes, while glycomics exhibits dynamic glycosylation patterns that regulate protein function, signaling, and cellular interactions [461,462].

**Figure 5:**
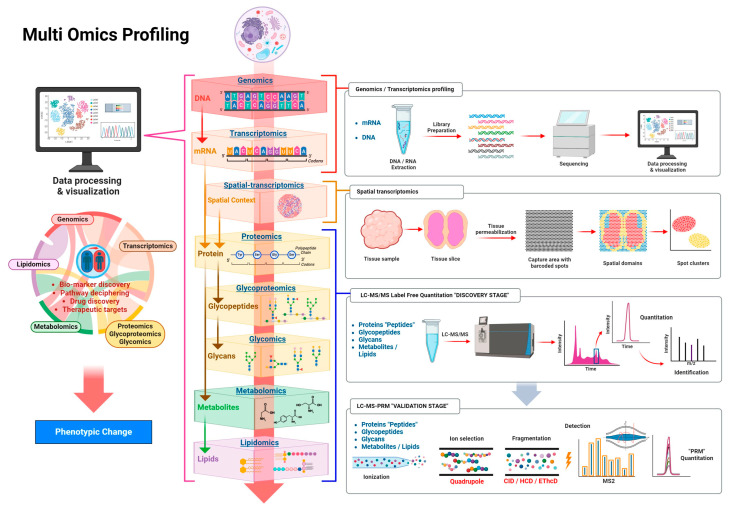
This figure illustrates a comprehensive multi-omics approach used to investigate biological systems by integrating genomics, transcriptomics, spatial transcriptomics, proteomics, glycoproteomics, glycomics, metabolomics, and lipidomics. Genomic and transcriptomic profiling begins with the extraction of DNA and mRNA, followed by library preparation, sequencing, and bioinformatic analysis to identify gene expression changes. Spatial transcriptomics adds tissue-level context by mapping gene expression across anatomical domains using barcoded capture arrays. Proteomics analyzes protein composition and abundance, while glycoproteomics and glycomics extend this by profiling glycopeptides and released glycans, respectively, to capture post-translational modifications critical to protein function and disease regulation. Metabolomics and lipidomics provide insights into small-molecule dynamics and membrane structure alterations. Data across all omics layers are analyzed using LC-MS/MS for quantification at the discovery stage and validated using parallel reaction monitoring (PRM) during targeted analysis. The integration of these omics platforms supports biomarker discovery, pathway elucidation, therapeutic target identification, and comprehensive interpretation of phenotypic changes resulting from molecular alterations. Adapted with permission from Gutierrez Reyes et al. [460].

Integrating omics datasets provides a comprehensive molecular network that elucidates disease mechanisms as depicted in Figure 5. Combining glycomics with proteomics enables the identification of specific protein glycoforms that serve as disease biomarkers. Meanwhile, integrating glycomics with metabolomics can reveal how glycosylation influences or is influenced by metabolic pathways, such as nucleotide-sugar biosynthesis or energy metabolism [463,464]. These approaches offer valuable insights in fields such as oncology, neurology, and immunology, where multi-omics integration has uncovered novel biomarkers, drug targets, and therapeutic pathways that remain undetected when using single-omics strategies alone. Aberrant glycosylation detected through glycomics is associated with changes in metabolic profiles and protein expression patterns, highlighting complex, multi-dimensional signatures linked to tumor progression and therapeutic response [465].

Despite its promising applications, integrated multi-omics faces significant technical and computational hurdles. Key challenges include data normalization and the need for robust bioinformatics tools. Nevertheless, advancements in high-throughput multi-omics integration are enhancing our understanding of disease complexity and individual variability, thereby advancing biomedical research.

### 5.3. Artificial Intelligence in Glycomics

Artificial intelligence (AI), particularly machine learning (ML), is increasingly recognized as a transformative approach in glycomics, offering solutions to longstanding challenges in glycan structural analysis, spectral interpretation, and biomarker discovery. ML algorithms, including support vector machines, random forests, k-nearest neighbors, and advanced deep learning architectures, have been successfully applied to predict glycosylation sites, classify glycan profiles, and annotate high-dimensional mass spectrometry datasets [466,467]. These data-driven approaches allow for the detection of subtle glycan alterations associated with various disease states such as cancer, neurodegenerative disorders, and viral infections [468,469]. Supervised learning has enabled the identification of disease-specific glycan signatures through training on well-characterized clinical datasets, while unsupervised methods such as clustering and dimensionality reduction techniques like principal component analysis, t-distributed stochastic neighbor embedding, and uniform manifold approximation and projection facilitate pattern recognition and subgroup stratification in exploratory studies. Deep learning models such as convolutional neural networks and recurrent neural networks are increasingly employed to decode complex glycomics spectra, extracting informative features from fragmentation patterns to improve structural characterization [470]. Additionally, AI plays a critical role in integrating glycomics with other omics platforms, including proteomics, transcriptomics, and metabolomics, thereby advancing systems-level understanding and supporting the development of multi-omics biomarker panels [471]. AI enhances analytical throughput, minimizes manual interpretation errors, and improves data reliability. As glycomics continues to produce increasingly large and complex datasets, the adoption of AI methodologies is expected to drive further innovation in data analysis, accelerate biomarker discovery, and facilitate the translation of glycomics research into clinical applications.

## 6. Conclusions

Glycosylation, as a ubiquitous and functionally diverse post-translational modification, plays a pivotal role in maintaining physiological homeostasis and mediating complex cellular processes such as protein folding, immune modulation, and intercellular communication. This review highlighted the structural complexity and regulatory capacity of glycans, highlighting their essential roles in health and their profound dysregulation across a spectrum of diseases, including cancer, neurodegenerative disorders, autoimmune conditions, cardiovascular pathologies, and infectious diseases. Altered glycosylation patterns not only serve as hallmarks of disease but also actively contribute to pathogenesis through mechanisms involving immune evasion, inflammation, synaptic dysfunction, and therapy resistance. The identification of disease-specific glycoforms and tumor-associated carbohydrate antigens (TACAs) presents significant opportunities for biomarker development and therapeutic targeting.

Advancements in glycomics technologies, such as mass spectrometry, glycan microarrays, and glycoengineering, have significantly enhanced our ability to profile the glycome with high specificity and resolution. The integration of single-cell glycomics, spatial glycoproteomics, and multi-omics approaches promises to further unravel the intricate roles of glycans in disease mechanisms and facilitate their clinical translation. Importantly, the interdisciplinary potential of glycomics is becoming increasingly evident, particularly through its integration with genomics, proteomics, and other omics approaches. Such multi-omics strategies promise to enhance precision medicine by providing a more comprehensive understanding of disease biology and enabling individualized therapeutic interventions. However, challenges remain in standardizing glycomic analyses and validating glyco-biomarkers across diverse populations and disease stages. Continued research in glycoscience, supported by interdisciplinary collaboration, will be critical to unlocking the full diagnostic, prognostic, and therapeutic potential of glycans. Ultimately, understanding and harnessing the glycome will enable the development of precision medicine strategies tailored to the glycosylation landscape of individual patients.

## Figures and Tables

**Table 1:** A summary of key established and emerging glycan biomarkers, including their associated diseases and corresponding clinical relevance.

Glycan and Glycoconjugates Biomarkers	Associated Diseases and Clinical Relevance
Tn antigen (GalNAcα1-O-Ser/Thr)	Found in breast, colon, lung, and esophageal cancers; indicates incomplete O-glycosylation and is a marker of immune evasion [22,172].
Sialyl-Tn (STn)	Detected in gastric, colon, and breast cancers; associated with metastasis and poor prognosis [24,129,173].
Carcinoembryonic Antigen (CEA)	Colorectal, lung, breast cancers; serum glycoprotein marker for cancer detection and recurrence monitoring [153,154].
Alpha-fetoprotein (AFP)	Liver cancer; diagnostic and prognostic serum marker [149,150].
Prostate-Specific Antigen (PSA)	Prostate cancer; widely used clinical tumor marker [155].
Sialyl Lewis X (sLeX)/Sialyl Lewis A (sLeA)	Expressed in pancreatic, colorectal cancers; promotes tumor cell adhesion and metastasis via selectin binding [131,145,185].
CA19-9 (Sialyl-Lewis A)	Pancreatic and gastrointestinal cancers; widely used serum tumor biomarker [156,184].
CA125 (MUC16)	Ovarian cancer; used for diagnosis and treatment monitoring [151,152].
Globo H	Highly expressed in breast, prostate, ovarian, and lung cancers; enhances tumor growth and immune evasion; target for cancer vaccines [176,182].
GD2 ganglioside	Present in neuroblastoma, glioma, and melanoma; a therapeutic target of monoclonal antibodies (e.g., dinutuximab) [130].
CA19-9 (sLeA)	Clinically used biomarker for pancreatic and GI cancers; reflects sialylated Lewis A expression [184].
High-mannose *N*-glycans	Elevated in congenital disorders of glycosylation (CDGs), cancer, and viral infections, and is used in diagnosis and pathogen recognition [35,291].
Bisecting GlcNAc	Increased in neurological disorders such as Alzheimer’s disease and cancer; affects tau glycosylation and immune signaling; detectable in CSF and serum [127,201,220].
Core fucose	Altered in SLE, RA, Alzheimer’s; affects IgG function and cancer immune evasion [21,73,269].
Hypogalactosylated IgG (G0 glycan)	Found in RA and SLE; promotes inflammation via complement activation and Fc receptor binding [30,31,251].
IgG sialylation	Decreased in Rheumatoid arthritis (RA), systematic lupus erythematosus (SLE), and Inflammatory Bowel Disease (IBD); correlates with pro-inflammatory activity and disease flares [254,267,270].
Polysialic acid (PolySia)	Reduced in Parkinson’s and Alzheimer’s disease; regulates synaptic plasticity and neuroinflammation [126,215].
GM1, GD1a (gangliosides)	Depleted in Parkinson’s and Alzheimer’s; linked to synaptic dysfunction and neurodegeneration [215].

## Data Availability

No new data were created or analyzed in this study. Data sharing does not apply to this article.
